# Expression of Ca^2+^-permeable two-pore channels rescues NAADP signalling in TPC-deficient cells

**DOI:** 10.15252/embj.201490009

**Published:** 2015-04-14

**Authors:** Margarida Ruas, Lianne C Davis, Cheng-Chang Chen, Anthony J Morgan, Kai-Ting Chuang, Timothy F Walseth, Christian Grimm, Clive Garnham, Trevor Powell, Nick Platt, Frances M Platt, Martin Biel, Christian Wahl-Schott, John Parrington, Antony Galione

**Affiliations:** 1 Department of Pharmacology, University of OxfordOxford, UK; 2 Center for Integrated Protein Science CIPS-M and Department of Pharmacy – Center for Drug Research, Ludwig-Maximilians-Universität MünchenMünchen, Germany; 3 Pharmacology Department, University of MinnesotaMinneapolis, MN, USA

**Keywords:** Ca^2+^, electrophysiology, endo-lysosome, NAADP, TPC

## Abstract

The second messenger NAADP triggers Ca^2+^ release from endo-lysosomes. Although two-pore channels (TPCs) have been proposed to be regulated by NAADP, recent studies have challenged this. By generating the first mouse line with demonstrable absence of both *Tpcn1* and *Tpcn2* expression *(Tpcn1/2*^−/−^*)*, we show that the loss of endogenous TPCs abolished NAADP-dependent Ca^2+^ responses as assessed by single-cell Ca^2+^ imaging or patch-clamp of single endo-lysosomes. In contrast, currents stimulated by PI(3,5)P_2_ were only partially dependent on TPCs. In *Tpcn1/2*^−/−^ cells, NAADP sensitivity was restored by re-expressing wild-type TPCs, but not by mutant versions with impaired Ca^2+^-permeability, nor by TRPML1. Another mouse line formerly reported as TPC-null likely expresses truncated TPCs, but we now show that these truncated proteins still support NAADP-induced Ca^2+^ release. High-affinity [^32^P]NAADP binding still occurs in *Tpcn1/2*^−/−^ tissue, suggesting that NAADP regulation is conferred by an accessory protein. Altogether, our data establish TPCs as Ca^2+^-permeable channels indispensable for NAADP signalling.

See also: **TJ Jentsch *et al*** (July 2015)

## Introduction

Ca^2+^ release from intracellular Ca^2+^ stores constitutes a universal cell signalling mechanism and is evoked by any of three principal Ca^2+^-mobilizing messengers: inositol 1,4,5-trisphosphate (IP_3_), cyclic ADP ribose (cADPR), and nicotinic acid adenine dinucleotide phosphate (NAADP) (Berridge *et al*, 2003). Recruited by extracellular stimuli as diverse as cell–cell contact and GPCR activation, NAADP has been implicated in processes such as fertilization, exocytosis, autophagy, cardiac and neural function, and cell differentiation (Galione, 2014). NAADP differs from IP_3_ and cADPR, which regulate IP_3_ receptors and ryanodine receptors, respectively, in the ER, by primarily targeting a different Ca^2+^ store (acidic endo-lysosomal organelles) (Churchill *et al*, 2002) and a different Ca^2+^-permeable channel (Galione, 2011). However, the molecular identity of this NAADP-regulated channel has proven controversial, with several candidate channel families being proposed without a common consensus being reached (Morgan *et al*, 2011; Guse, 2012; Marchant & Patel, 2013).

Therefore, the proposal that the two-pore channel (TPC) family are Ca^2+^-permeable channels regulated by NAADP was a promising development (Brailoiu *et al*, 2009; Calcraft *et al*, 2009; Zong *et al*, 2009); TPCs are endo-lysosomal channels with homologies to TRP (one-domain) and Ca_V_ (four-domain) channels, with a predicted intermediate two-domain structure that probably assembles as dimers (Rietdorf *et al*, 2011; Churamani *et al*, 2012). Although a three-gene family, several species, including mice and humans, only have *Tpcn1* and *Tpcn2* genes.

TPCs are emerging as physiologically important channels mediating NAADP signalling in diverse contexts, for example cell differentiation, angiogenesis, immune cell signalling, smooth muscle contraction, autophagy, and cardiovascular and liver physiology (Aley *et al*, 2010; Tugba Durlu-Kandilci *et al*, 2010; Esposito *et al*, 2011; Davis *et al*, 2012; Lu *et al*, 2013; Zhang *et al*, 2013; Favia *et al*, 2014; Grimm *et al*, 2014). Moreover, TPCs are the only known Ca^2+^-release channels in plants, where they mediate long-range Ca^2+^ waves (Choi *et al*, 2014).

Several lines of evidence from different groups support TPCs as NAADP-regulated channels with many of the expected properties: manipulation of TPC expression (by overexpression, RNAi or gene disruption) paralleled NAADP-dependent responses in multiple systems (Morgan & Galione, 2014), and NAADP-dependent currents were observed with both over-expressed TPCs and affinity-purified TPCs in lipid bilayers (Pitt *et al*, 2010, 2014; Rybalchenko *et al*, 2012), with single-organelle planar patch-clamp (Schieder *et al*, 2010) or with cells in which TPCs were re-directed to the plasma membrane (Brailoiu *et al*, 2010; Yamaguchi *et al*, 2011; Jha *et al*, 2014). Furthermore, recent studies have suggested that TPCs may not bind NAADP directly but rather require an accessory protein (Lin-Moshier *et al*, 2012; Walseth *et al*, 2012a,b) that co-immunoprecipitates with TPCs (Ruas *et al*, 2010; Walseth *et al*, 2012a).

Against this compelling body of evidence, recent papers challenged the status of TPCs as NAADP-regulated Ca^2+^-permeable channels by proposing that TPCs are instead Na^+^-selective channels activated by the phosphoinositide lipid PI(3,5)P_2_ (phosphatidylinositol 3,5-bisphosphate) but not by NAADP (Wang *et al*, 2012; Cang *et al*, 2013). Their conclusions were drawn from the use of a mouse line designed to knockout both *Tpcn1* and *Tpcn2* expression in combination with conventional patch-clamp of endo-lysosomes and Ca^2+^ imaging. However, whether these mice are *bona fide* TPC-null is open to debate as they have the potential to express ≥ 91% of the full-length TPC sequences (Morgan & Galione, 2014; Ruas *et al*, 2014).

In view of these conflicting findings, and given the emerging importance of NAADP and TPCs in cell signalling, it is a matter of urgency to rigorously define the relationship between TPCs and NAADP-regulated Ca^2+^ release. Therefore, we have generated and fully characterized a new transgenic mouse line with a demonstrable absence of both *Tpcn1* and *Tpcn2* expression. This has allowed us to examine for the first time the effect of loss of endogenous TPC1 and TPC2 proteins on single-cell Ca^2+^ release or native currents from single endo-lysosomes and the effects of their re-expression. Our data reaffirm that TPCs are essential for NAADP-induced Ca^2+^ signalling and NAADP-stimulated endo-lysosomal Ca^2+^-permeable currents, but are not essential for PI(3,5)P_2_-mediated currents.

## Results

### Generation of *Tpcn1/2*^−/−^ mice with demonstrable lack of *Tpcn1* and *Tpcn2* expression

**Figure 1:**
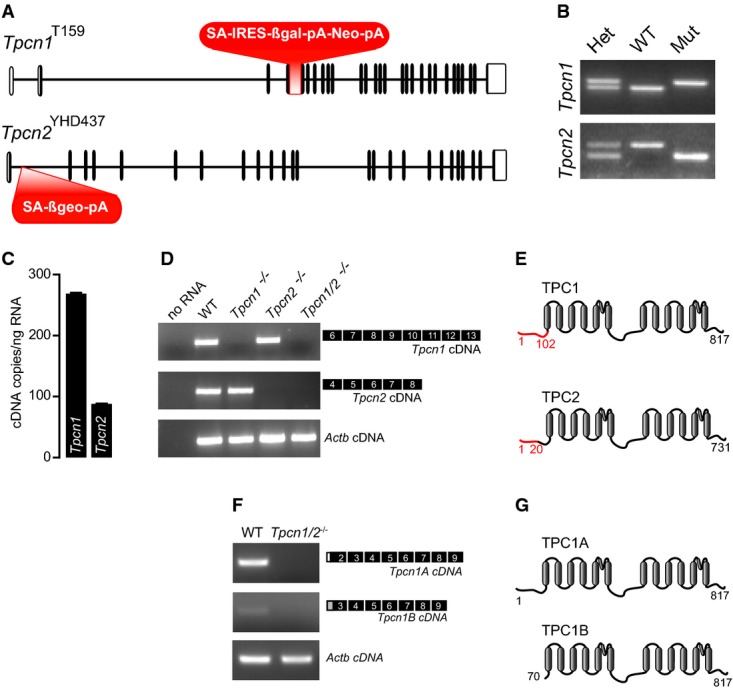
Characterization of MEFs derived from *Tpcn* knockout mice

We generated a mouse line carrying *Tpcn1*^T159^ (Ruas *et al*, 2014) and *Tpcn2*^YHD437^ (Calcraft *et al*, 2009) mutant alleles (Fig1A and B) and have prepared mouse embryonic fibroblasts (MEF) from *Tpcn1*^T159^/*Tpcn2*^YHD437^ animals. RT–qPCR analysis revealed that MEFs express both *Tpcn1* and *Tpcn2* (Fig1C); no detectable levels of *Tpcn1* or *Tpcn2* mRNAs were observed in MEFs from *Tpcn1*^T159^/*Tpcn2*^YHD437^ animals, including a newly identified *Tpcn1B* isoform arising from an alternative promoter (Ruas *et al*, 2014) (Fig1D–G). Expression from the *Tpcn* mutant alleles in *Tpcn1*^T159^/*Tpcn2*^YHD437^ animals is predicted to result in production of only small portions of the N-terminal tails of the respective TPC proteins (Fig1E), corresponding to only the first 102 (for TPC1) or 20 (for TPC2) amino acid residues (≤ 12% of the full-length sequence). This contrasts with a mutant mouse line (developed by D. Ren and referred to hereafter as *Tpcn1/2*^Dren^) used in recent studies in which ≥ 91% of the full-length TPC sequence could be still expressed, that is 748 (for TPC1; equivalent to TPC1B) or 682 (for TPC2) amino acid residues (Wang *et al*, 2012; Cang *et al*, 2013) (see below and Fig 7A).

Gene structure of *Tpcn1*^T159^ and *Tpcn2*^YHD^^437^ alleles in transgenic mice. Exons are represented as vertical segments (UTRs, unfilled boxes); knockout and gene trap cassettes are represented in red. Splice acceptor (SA), internal ribosomal entry site (IRES), β-galactosidase gene (βgal), neomycin resistance gene (Neo), β-galactosidase/neomycin resistance chimeric gene (βgeo), polyadenylation signal (pA).

Genotyping results for homozygote wild-type (WT), homozygote mutant *Tpcn1*^T159^ or *Tpcn2*^YHD^^437^ (Mut), and heterozygote animals (Het).

RT–qPCR analysis of absolute levels of *Tpcn1* and *Tpcn2* transcripts in WT MEFs. *Tpcn1*/*Tpcn2* ratio of expression corresponds to 3.0; *n *= 6; mean ± SEM.

RT–PCR analysis of *Tpcn1* and *Tpcn2* expression in MEFs from WT or homozygote transgenic embryos. Amplified cDNAs correspond to exons shown in black. Expression of *Actb* was used as a control.

Two-domain organization of TPC1 and TPC2 proteins showing transmembrane helices (grey) and amino acid residues (numbers). Predicted residual expression of TPC proteins from transgenic animals is represented in red.

RT–PCR analysis of *Tpcn1A* and *Tpcn1B* expression in MEFs from WT or *Tpcn1/2*^−/−^ embryos. Amplified cDNAs correspond to the exons shown in black including isoform-specific 5′-UTRs (white box for *Tpcn1A* and grey box for *Tpcn1B*). Expression of *Actb* was used as a control.

TPC1 protein variants expressed from *Tpcn1A* and *Tpcn1B* transcripts.

Source data are available online for this figure.

These results indicate unequivocally that the mice we have generated have knocked-out expression for both of the *Tpcn* genes, which we therefore refer to as *Tpcn1/Tpcn2* double knockout (*Tpcn1/2*^−/−^).

### NAADP induces Ca^2+^ release from acidic Ca^2+^ stores

**Figure 2:**
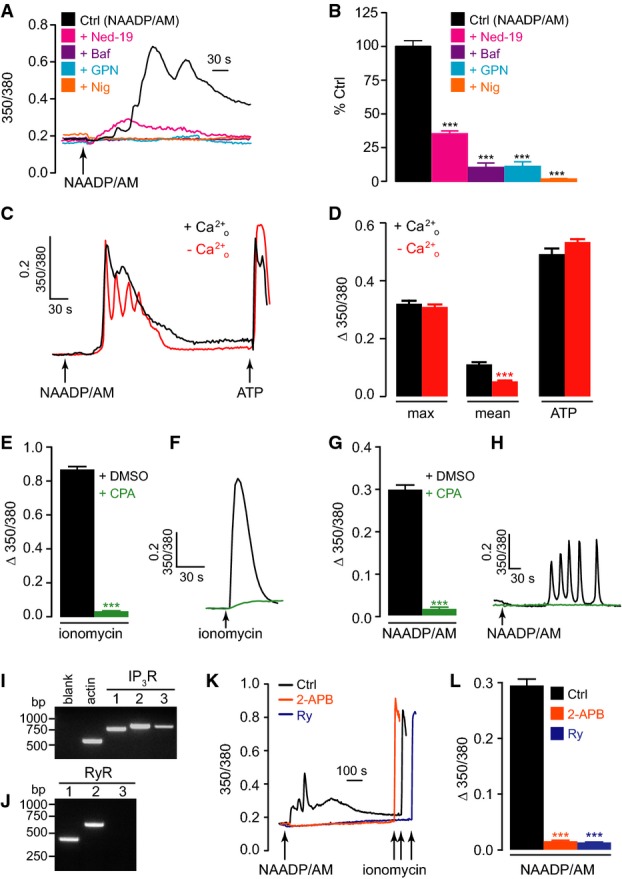
NAADP induces Ca^2+^ release from acidic Ca^2+^ stores

MEFs were analysed for their ability to respond to NAADP. Cytosolic Ca^2+^ was monitored with fura-2, and NAADP was bath-applied as its cell-permeant ester form, NAADP/AM. In wild-type MEFs, NAADP/AM evoked robust Ca^2+^ signals which were inhibited by pre-treatment with bafilomycin A1, GPN, and nigericin, agents that deplete acidic Ca^2+^ stores, and by the NAADP antagonist *trans*-Ned-19 (Fig2A and B). This is consistent with NAADP releasing Ca^2+^ from endo-lysosomes.

A, B Representative single-cell Ca^2+^ traces showing 350/380 ratios of fura-2 fluorescence (A) and maximum Ca^2+^ changes (B) upon addition of 10 μM extracellular NAADP/AM in WT MEFs, which were blocked by pre-incubation with 10 μM *trans*-Ned-19 (Ned-19; 45 min), 1 μM bafilomycin A1 (Baf; 45 min), 5 μM nigericin (Nig; 30 min), or 200 μM GPN (5 min); control (Ctrl) was pre-incubated with DMSO (vehicle); *n* = 121–272; ***P < 0.001 relative to control using the ANOVA-Tukey test.

C, D Ca^2+^ signals with 10 μM NAADP/AM in 1.8 mM extracellular Ca^2+^ (+ Ca^2+^_o_) or Ca^2+^-free medium containing 100 μM EGTA (− Ca^2+^_o_) in WT MEFs. (C) Representative single-cell fura-2 Ca^2+^ traces upon addition of 10 μM NAADP/AM and 100 μM ATP. (D) Maximum Ca^2+^ changes (max) and mean Ca^2+^ release over a period of 300 s post-addition of 10 μM NAADP/AM;*n* = 233–385 cells; ***P < 0.001 relative to + Ca^2+^_o_ using an unpaired *t*-test.

E–H Cells treated with 200 μM CPA or 0.1% DMSO in medium + Ca^2+^_o_ for 50 min. Cells were then briefly washed and maintained in Ca^2+^-free medium (+100 µM EGTA) in which they were stimulated with 2 μM ionomycin (E, F) or 10 μM NAADP/AM (G, H). Maximum Ca^2+^ changes (E, G) and representative single-cell fura-2 Ca^2+^ traces (F, H); *n* = 49–148 cells; ***P < 0.001 relative to DMSO control, using the unpaired *t*-test.

I, J MEFs express all three IP_3_ receptor subtypes (IP_3_R 1–3) and ryanodine receptor (RyR) types 1 and 2, detected by RT–PCR analysis. Blank refers to no RNA. Positive control for expression for RyR type 3 is shown in Supplementary Fig S1.

K, L Cells treated with 2 μM 2-APB, 20 μM ryanodine, or 0.1% DMSO prior to application of 10 μM NAADP/AM. Representative single-cell fura-2 Ca^2+^ traces (K) and maximum Ca^2+^ changes (L); *n* = 142–374; ***P < 0.001 relative to control using the unpaired *t*-test.

Data information: Error bars represent SEM. See also Supplementary Fig S1. Source data are available online for this figure.

To ascertain whether Ca^2+^ influx contributed to the NAADP response, we repeated experiments in Ca^2+^-free medium (Fig2C and D). The maximum amplitude of the NAADP-induced Ca^2+^ release was unaffected by removing external Ca^2+^ confirming that this early phase of the response is entirely due to intracellular Ca^2+^ release. That the mean Ca^2+^ response was, overall, somewhat reduced in Ca^2+^-free medium (Fig2C and D) suggested that Ca^2+^ influx played a role in sustaining the response but that it was not essential for NAADP action.

The long-standing “trigger hypothesis” describes NAADP as a provider of an initial “trigger” of Ca^2+^ that is subsequently amplified by Ca^2+^ release from the ER by virtue of the Ca^2+^ sensitivity of the IP_3_ receptor (IP_3_R) or ryanodine receptor (RyR), that is, Ca^2+^-induced Ca^2+^ release (CICR). We confirmed the co-involvement of the ER in several ways, first by depleting the ER with the Ca^2+^-ATPase inhibitor cyclopiazonic acid (CPA) (Fig2E and F), which abrogated NAADP/AM responses (Fig2G and H). Given that IP_3_R1–3 and RyR1–2 were all detected by RT–PCR in our WT MEFs (Fig2I and J and Supplementary Fig S1), we tested which ER channel families were functionally important; IP_3_R and RyR blockade with 2-APB (2-aminoxydiphenylborate) and ryanodine, respectively, abolished NAADP/AM-stimulated Ca^2+^ signals (Fig2K and L). NAADP-induced responses in the well-characterized pancreatic acinar cell exhibit a similar pharmacology (Cancela *et al*, 1999). Together with the fact that NAADP required acidic Ca^2+^ stores (Fig2A and B), these data are consistent with the trigger hypothesis whereby NAADP provides the trigger Ca^2+^ from acidic stores that is subsequently amplified by IP_3_Rs and/or RyRs on the ER (Churchill & Galione, 2000).

### TPC knockout abrogates NAADP-induced Ca^2+^ signals

**Figure 3:**
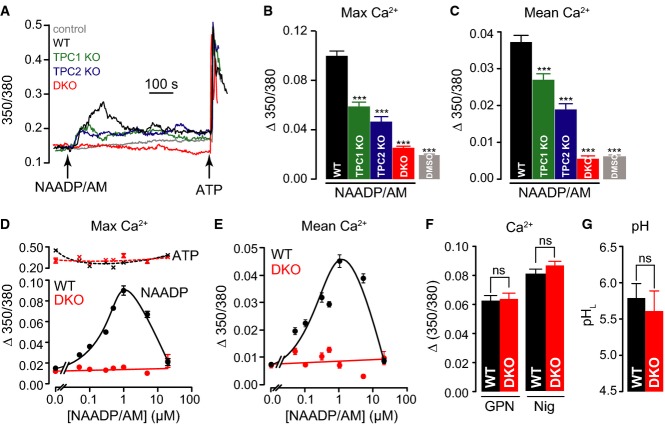
TPC knockout abrogates NAADP-induced Ca^2+^ signals

Using MEFs obtained from TPC knockout animals, we tested the requirement of TPCs for NAADP-induced Ca^2+^ signals. In WT MEFs, NAADP/AM evoked robust Ca^2+^ signals (Fig3A–E) that were approximately 40% of the amplitude of that evoked by the purinergic agonist ATP (Fig3D). In single-knockout MEFs lacking either TPC1 or TPC2, the NAADP responses were still present but significantly reduced in terms of the maximum amplitude or the mean Ca^2+^ signal (Fig3A–C); TPC2 knockout also affected NAADP responses in macrophages derived from adult mice (Supplementary Fig S2), a cell type in which it was recently argued that TPCs were NAADP insensitive (Wang *et al*, 2012). Critically, in *Tpcn1/2*^−/−^ MEFs, NAADP responses were eliminated while ATP responses remained robust (Fig3A–E). Note that the effects of TPC ablation cannot be due to altered Ca^2+^ influx because the peak responses to NAADP are independent of Ca^2+^ entry (Fig2D).

A–C Representative single-cell fura-2 Ca^2+^ traces (A), maximum Ca^2+^ changes (B), and mean Ca^2+^ release over a period of 500 s (C), post-addition of 10 μM NAADP/AM to wild-type (WT), *Tpcn1*^−/−^ (TPC1 KO), *Tpcn2*^−/−^ (TPC2 KO), and *Tpcn1/2*^−/−^ (DKO) MEFs. Control corresponds to WT cells treated with DMSO;*n* = 384–621; ***P < 0.001 relative to WT using the ANOVA–Tukey test.

D, E Maximum amplitude (D) and mean Ca^2+^ (E) of the responses to different NAADP/AM concentrations in WT and DKO cells. The subsequent maximum response to 100 μM ATP (cf. (A)) after each NAADP/AM concentration is also plotted (D). 1 μM NAADP/AM induced a maximal Ca^2+^ peak corresponding to 39 ± 3% of the 100 μM ATP response; *n* = 41–105.

F Maximum Ca^2+^ responses to 200 μM GPN or 10 μM nigericin; *n* = 111–285; *P* > 0.05 (ns) relative to WT using the ANOVA–Tukey test.

G Endo-lysosomal luminal pH (pH_L_) by endocytosed fluorescently labelled dextrans in primary MEFs determined by single-cell measurements; *n* = 105 for WT or DKO.

Data information: Error bars represent SEM. See also Supplementary Fig S2 and Fig S3.

Next, we checked whether TPC disruption simply shifted the NAADP concentration–response curve; in WT cells, addition of NAADP/AM over a wide range of concentrations produced the bell-shaped curve (Fig3D and E), that is a characteristic of mammalian NAADP-regulated Ca^2+^ signalling (Galione, 2011), and although *Tpcn1/2*^−/−^ cells responded well to ATP, there was no response to NAADP at any concentration tested (Fig3D and E).

Finally, we checked Ca^2+^ storage and luminal pH (pH_L_) within the endo-lysosomal system, either of which could potentially affect NAADP-induced Ca^2+^ release (Pitt *et al*, 2010, 2014; Schieder *et al*, 2010; Rybalchenko *et al*, 2012; Wang *et al*, 2012). The lack of NAADP-induced Ca^2+^ release in *Tpcn1/2*^−/−^ cells was not due to an absence of releasable Ca^2+^ because lysosomotropic agents evoked similar Ca^2+^ signals when compared to WT cells (Fig3F). Similarly, the pH_L_ measured across the entire endo-lysosomal system was unaffected as determined by ratiometric pH_L_ recordings (Fig3G and Supplementary Fig S3).

Taken together, these data indicate that TPC1 and TPC2 contribute to NAADP-evoked Ca^2+^ signalling and that removing both TPCs eradicates the ability of cells to respond to NAADP by directly affecting Ca^2+^ release, not endo-lysosomal Ca^2+^ storage or pH_L_.

### TPCs are required for NAADP-evoked endo-lysosomal currents

**Figure 4:**
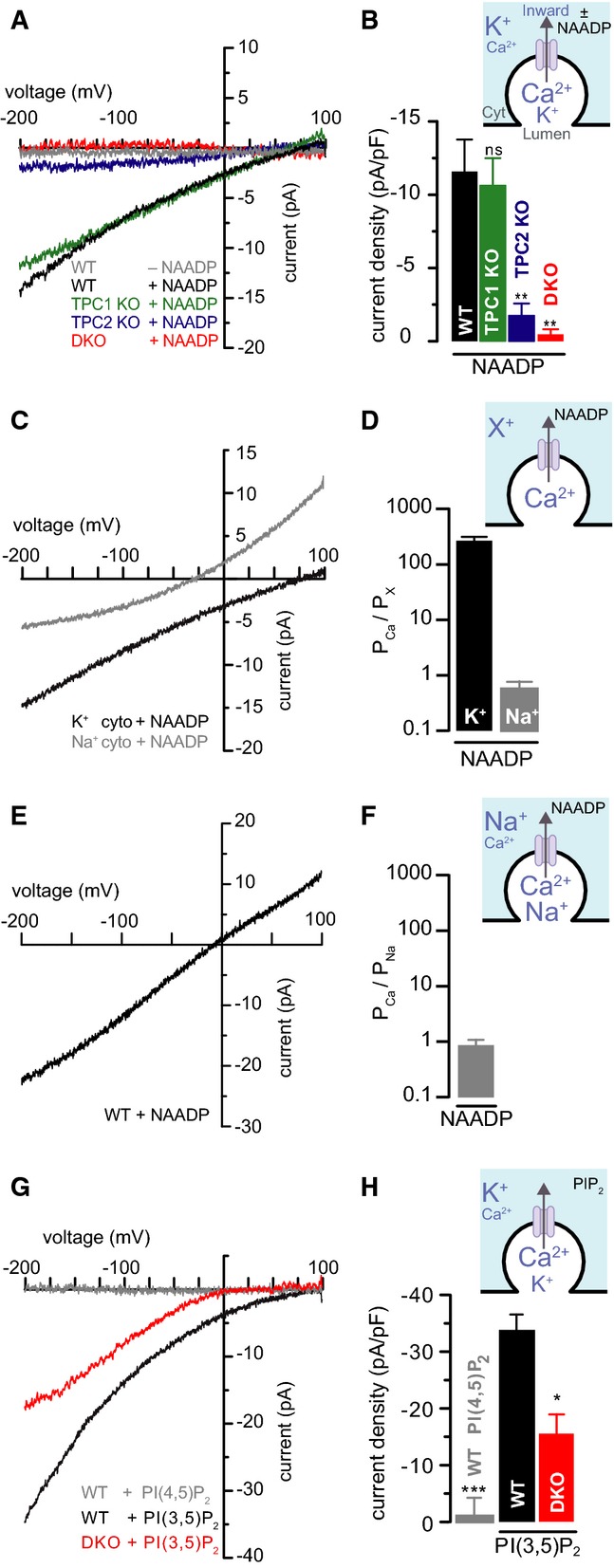
NAADP-stimulated Ca^2+^ currents are absent in TPC-null MEFs

Although the above data suggest that TPCs are essential for NAADP-induced Ca^2+^ signals, they do not explicitly demonstrate the activation of Ca^2+^-permeable channels on endo-lysosomes by NAADP. Therefore, we monitored native currents by planar patch-clamp of single whole endo-lysosomes swollen with vacuolin-1 and purified from WT or TPC knockout MEFs; importantly, such swelling does not affect NAADP-induced Ca^2+^ signalling (Supplementary Fig S4). In the presence of K^+^ and Ca^2+^ (but in the absence of Na^+^), cytosolic nanomolar concentrations of NAADP stimulated an inward current (lumen to cytoplasm) (Fig4A and B) with a reversal potential of +75 ± 7 mV, in WT MEFs. This is consistent with Ca^2+^ being the major permeant ion under these conditions (equilibrium potentials, *E*_K_ = −16 mV, *E*_Ca_ = +73 mV). Importantly, NAADP-induced currents were undetectable in similar preparations from *Tpcn1/2*^−/−^ and *Tpcn2*^−/−^ cells, while they were still present (reversal potential of +75 ± 4 mV) in preparations from *Tpcn1*^−/−^ cells (Fig4A and B). This implicates TPCs as the predominant Ca^2+^-permeant channels in endo-lysosomes regulated by NAADP, but largely carried by TPC2 in MEFs under our conditions.

A, B Single-lysosome currents from wild-type (WT), *Tpcn1*^−/−^ (TPC1 KO), *Tpcn2*^−/−^ (TPC2 KO), and *Tpcn1/2*^−/−^ (DKO) MEFs in the presence or absence of cytosolic NAADP (50 nM); solutions contained Ca^2+^ (cytosol: 0.2 mM; lumen: 60 mM) plus K^+^ (cytosol: 130 mM; lumen: 70 mM). Inward currents are defined as lumen-to-cytosol. (A) Representative current–voltage (I–V) curves from single isolated lysosomes. (B) Population data were measured at −200 mV from (A); *n* = 5–6; ns, *P* > 0.05, **P < 0.01 relative to WT using the ANOVA-Tukey test.

C, D NAADP (50 nM)-evoked single-lysosome currents from WT MEFs under bi-ionic conditions: 160 mM monovalent “X^+”^ (either K^+^ or Na^+^) in the cytosol and 107 mM Ca^2+^ in the lumen. (C) Representative I–V curves from isolated lysosomes. (D) Population data of the relative Ca^2+^/monovalent permeability ratios; *n* = 9–11.

E, F Single-lysosome currents from WT MEFs in the presence of cytosolic NAADP (50 nM); solutions contained Ca^2+^ (cytosol: 0.2 mM; lumen: 61 mM) and Na^+^ (cytosol: 160 mM; lumen: 70 mM). (E) Representative I–V curve from single lysosomes derived from WT MEFs. (F) Population data of the relative Ca^2+^/Na^+^ permeability ratios; *n* = 6.

G, H Single-lysosome currents from WT or DKO MEFs in the presence of cytosolic PI(3,5)P_2_ (10 μM) or PI(4,5)P_2_ (10 μM); solutions contained Ca^2+^ (cytosol: 0.2 mM; lumen: 60 mM) plus K^+^ (cytosol: 130 mM; lumen: 70 mM). (G) Representative I–V curves from single lysosomes derived from WT or DKO MEFs. (H) Population data were measured at −200 mV from (G); *n* = 3–4; ***P < 0.001, *P < 0.05 relative to WT/PI(3,5)P_2_ using Student's *t*-test.

Data information: Error bars represent SEM. See also Supplementary Fig S4.

In view of recent proposals that TPCs also conduct Na^+^ (Wang *et al*, 2012; Cang *et al*, 2013, 2014; Boccaccio *et al*, 2014; Jha *et al*, 2014; Pitt *et al*, 2014), we quantified the ion selectivity of TPCs in our preparation, by performing experiments under bi-ionic conditions (luminal Ca^2+^, cytosolic monovalent). With cytosolic K^+^, the reversal potential was +76 ± 2 mV which equates to a *P*_Ca_/*P*_K_ permeability ratio of 268 ± 47 (Fig4C and D). By contrast, with Na^+^ as the monovalent ion, the reversal potential was −22 ± 5 mV which equates to a *P*_Ca_/*P*_Na_ permeability ratio of 0.57 ± 0.19 (Fig4C and D).

Additionally, we measured the relative Ca^2+^ permeability in the presence of luminal Na^+^. Because seal formation requires luminal Ca^2+^, currents were necessarily recorded with both Ca^2+^ and Na^+^ in the lumen. Under these conditions, NAADP stimulated an inward current with a reversal potential of −3.8 ± 2.9 mV (equilibrium potentials, *E*_Na_ = −21 mV, *E*_Ca_ = +73 mV), which equates to a permeability ratio *P*_Ca_/*P*_Na_ of 0.86 ± 0.22 (Fig4E and F). Therefore, the permeability ratio was the same irrespective of whether Na^+^ was just cytosolic or on both sides of the membrane.

These results demonstrate that the permeability of TPCs to Na^+^ and Ca^2+^ is of the same order of magnitude, thus differing from the proposal that TPCs are highly Na^+^-selective channels (Wang *et al*, 2012; Cang *et al*, 2013, 2014). In other words, the NAADP-stimulated current displays a rank order of selectivity of Na^+^ ≥ Ca^2+^ ≫ K^+^. Furthermore, these results suggest that NAADP-induced Ca^2+^ currents are mediated by endogenous TPCs and not by other proposed NAADP-activated endo-lysosomal channels such as TRPML1 (Zhang *et al*, 2009) or TRPM2, the latter being activated by NAADP at much higher concentrations [EC_50_ 100–730 μM (Lange *et al*, 2008)].

The endo-lysosome-specific lipid, PI(3,5)P_2_, has been reported to regulate both TRPML1 (Dong *et al*, 2010) and TPC channels (Wang *et al*, 2012; Cang *et al*, 2013, 2014; Boccaccio *et al*, 2014; Grimm *et al*, 2014; Jha *et al*, 2014; Pitt *et al*, 2014). In WT endo-lysosomes, robust Ca^2+^ currents (reversal potential +70 ± 10 mV) were stimulated by PI(3,5)P_2_, whereas PI(4,5)P_2_ was without effect (Fig4G and H). Interestingly, PI(3,5)P_2_-stimulated currents were still seen in *Tpcn1/2*^−/−^ endo-lysosomes, but were reduced (Fig4G and H), which suggests that both TPC-dependent and TPC-independent currents are modulated by the lipid; indeed, the residual TPC-independent currents unmasked in *Tpcn1/2*^−/−^ endo-lysosomes were markedly inwardly rectifying with a reversal potential of −6 ± 13 mV and therefore consistent with TRPML1-mediated K^+^ currents (*E*_K_ = −16 mV) (Dong *et al*, 2010).

Together, these data indicate that while NAADP-induced endo-lysosomal currents are wholly dependent on TPCs, PI(3,5)P_2_-induced currents can also be mediated by other endo-lysosomal channels as may be predicted for a permissive lipid endo-lysosomal channel modulator (Cang *et al*, 2014).

### TPC expression rescues NAADP-induced Ca^2+^ release in *Tpcn1/2*^−/−^ MEFs

**Figure 5:**
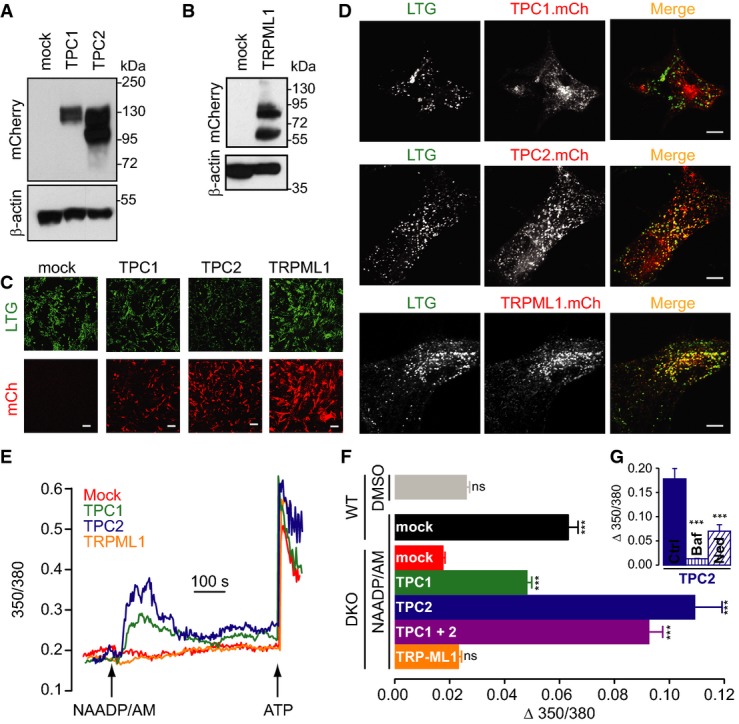
Re-expression of TPC1 and TPC2, but not TRPML1, recovers NAADP-induced Ca^2+^ release in DKO MEFs

To confirm that the loss of NAADP responsiveness in *Tpcn1/2*^−/−^ MEFs was due to the specific lack of TPCs, we restored expression of TPCs and assessed NAADP-induced Ca^2+^ responses. Thus, *Tpcn1/2*^−/−^ MEFs were transduced with lentiviruses for expression of either mouse TPC1 or TPC2 tagged with a C-terminal mCherry. Immunoblot analysis confirmed that transduction resulted in expression of TPC1 and TPC2 (Fig5A) and live-cell fluorescence verified that they were expressed in all cells (Fig5C and Supplementary Fig S5) with the expected pattern of localization; while TPC1 shows a more modest co-localization with LysoTracker Green and consistent with recycling endosomes (Calcraft *et al*, 2009; Ruas *et al*, 2014), TPC2 shows a strong co-localization with LysoTracker Green, indicative of late endosomal/lysosomal localization, as confirmed by other endo-lysosomal markers (Fig5D and Supplementary Fig S6).

A, B Immunoblotting analysis of *Tpcn1/2*^−/−^ (DKO) MEFs expressing mCherry-tagged mouse TPC1 and TPC2 (mock, empty vector) (A) or mouse TRPML1 (B). The top half of the blot was probed for mCherry and the bottom half for β-actin as a loading control.

**Figure 6:**
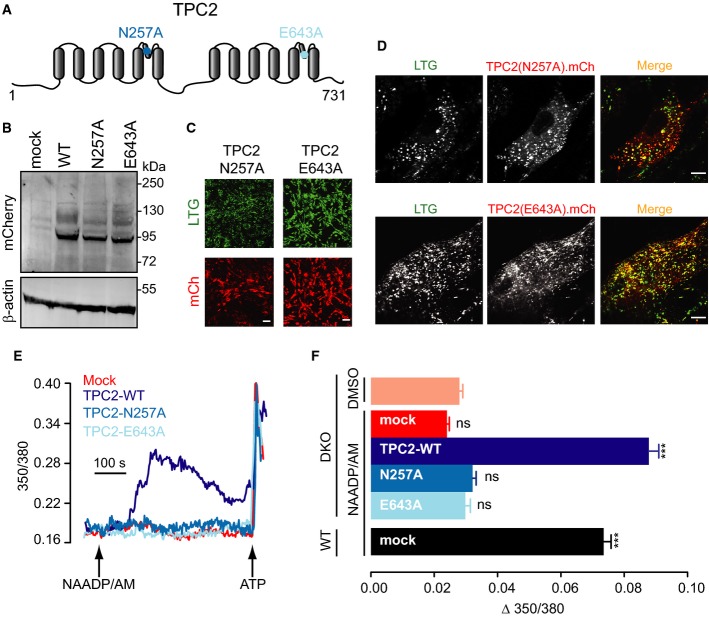
N-terminal truncated forms of TPC1 or TPC2 rescue NAADP-induced Ca^2+^ release in DKO MEFs

**Figure 7:**
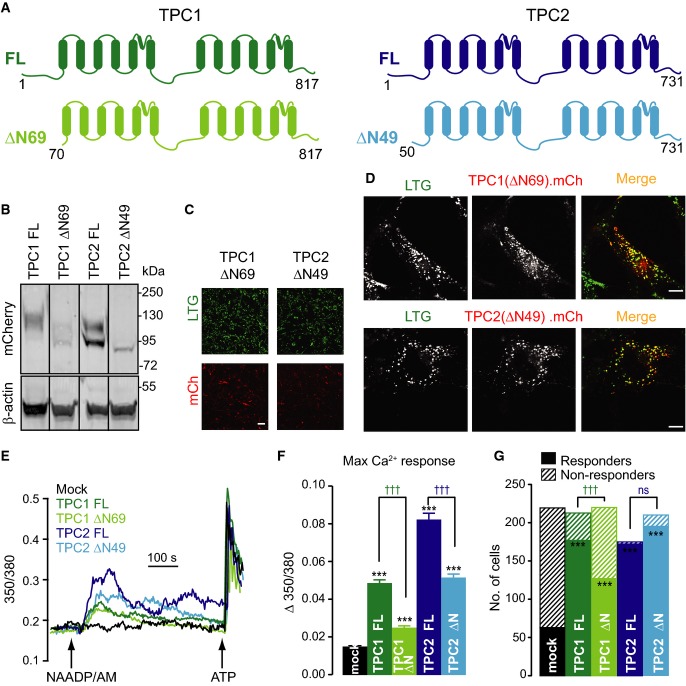
Expression of TPC2 pore mutants fails to restore NAADP-induced Ca^2+^ release in DKO MEFs

C, D Live-cell imaging of MEF cells expressing mCherry-tagged proteins (LTG, LysoTracker Green signal; mCh, mCherry signal). Scale bar, 100 μm (C; larger images are shown in Supplementary Fig S5) or 10 μm (D). Images in (C) were taken with the same acquisition settings as in Figs6C and 7C.

E, F Representative fura-2 Ca^2+^ traces from DKO MEFs expressing mCherry-tagged proteins (E) and maximum Ca^2+^ responses induced by 10 μM NAADP/AM (F); DMSO represents control for NAADP/AM addition; *n* = 137–468; ***P < 0.001, ns, *P* > 0.05 relative to DKO/mock using the ANOVA–Tukey test.

G Maximum Ca^2+^ responses induced by 10 μM NAADP/AM in TPC2-transduced DKO MEFs are inhibited by pre-incubation with 1 μM bafilomycin A1 (Baf; 45 min) or 10 μM *trans*-Ned-19 (Ned-19; 45 min); *n* = 63–113; ***P < 0.001 relative to control (0.1% DMSO) using the ANOVA–Tukey test.

Data information: Error bars represent SEM. See also Supplementary Fig S5 and Fig S6. Source data are available online for this figure.

A Schematic representation of TPC1 and TPC2 proteins corresponding to full-length (FL) and N-terminal truncations (ΔN) predicted to be expressed in the mutant *Tpcn1/2*^Dren^ mice used in Cang *et al* (2014, 2013) and Wang *et al* (2012). Transmembrane helices are represented by vertical blocks, and numbers represent amino acid residues.

B Immunoblotting analysis of *Tpcn1/2*^−/−^ (DKO) MEFs expressing mCherry-tagged mouse TPC1 and TPC2 and full-length (FL) and N-terminal truncations (ΔN). Blot was probed for mCherry and for β-actin as a loading control. Further immunoblots from PNGase F-treated samples are shown in Supplementary Fig S7.

C, D Live-cell imaging of MEF cells expressing mCherry-tagged proteins (LTG, LysoTracker Green signal; mCh, mCherry signal). Scale bar, 100 μm (C; larger images are shown in Supplementary Fig S5) or 10 μm (D). Images in (C) were taken under the same acquisition parameters as in Figs5C and 6C.

E, F Representative fura-2 Ca^2+^ traces from DKO MEFs expressing mCherry-tagged proteins (mock, empty vector) (E) and maximum Ca^2+^ responses induced by 10 μM NAADP/AM (F); *n* = 171–224; ***P < 0.001 relative to mock whereas ^†††^*P* < 0.001 comparing FL to ΔN using the ANOVA–Tukey test.

G Comparison of number of responding cells to NAADP/AM treatment for each set of transduced DKO MEF cells. Only a cell showing a maximum NAADP/AM-induced Ca^2+^ response greater than the standard deviation of the basal 350/380 ratio for its set was considered as a responder; ***P < 0.001 relative to mock whereas ^†††^*P* < 0.001 comparing FL to ΔN using contingency tables.

Data information: Error bars represent SEM. See also Supplementary Fig S5 and Fig S7. Source data are available online for this figure.

A Schematic representation of TPC2 protein with highlighted pore mutations.

B Immunoblotting analysis of *Tpcn1/2*^−/−^ (DKO) MEFs expressing mCherry-tagged mouse wild-type TPC2 (WT) and pore mutants N257A and E643A (mock, empty vector). Blot was probed for mCherry and for β-actin as a loading control.

C, D Live-cell imaging of MEF cells expressing mCherry-tagged TPC2 mutant pore proteins (LTG, LysoTracker Green signal; mCh, mCherry signal). Scale bar, 100 μm (C; larger images are shown in Supplementary Fig S5) or 10 μm (D). Images in (C) were taken with the same acquisition settings as in Figs5C and 7C.

E, F Representative fura-2 Ca^2+^ traces from DKO MEFs expressing mCherry-tagged TPC2 proteins (E) and maximum Ca^2+^ responses induced by 10 μM NAADP/AM (F); *n* = 311–413; ***P < 0.001, ns, *P* > 0.05 relative to DKO/DMSO using the ANOVA–Tukey test.

Data information: Error bars represent SEM. See also Supplementary Fig S5. Source data are available online for this figure.

We then examined NAADP-induced Ca^2+^ signals in *Tpcn1/2*^−/−^ MEFs after re-expression of TPC proteins and compared them to responses in mock-transduced cells. We observed no NAADP-induced Ca^2+^ signals in mock-transduced *Tpcn1/2*^−/−^ cells, comparable to DMSO alone in WT cells (the vehicle control for NAADP/AM; Fig5E and F). Strikingly, re-expression of either TPC1 or TPC2 in *Tpcn1/2*^−/−^ MEFs restored NAADP responsiveness, with TPC2 being the more efficient (Fig5E and F) and restoring Ca^2+^ responses beyond those observed in mock-transduced WT cells (Fig5F). Co-expression of both TPCs had no greater effect than TPC2 alone (Fig5F). Importantly, the Ca^2+^ responses observed in TPC2-rescued cells exhibit the expected pharmacology: they were inhibited by bafilomycin A1 and *trans*-Ned-19 (Fig5G). Additionally, the rescue was specific to TPCs because expression of the Ca^2+^-permeable endo-lysosomal TRPML1 in *Tpcn1/2*^−/−^ MEFs (Fig5B–F) failed to have any effect, further arguing against its being an NAADP-regulated channel (Pryor *et al*, 2006; Yamaguchi *et al*, 2011).

### Pore-mutant TPCs fail to rescue NAADP-induced Ca^2+^ release

To ascertain whether TPCs rescue NAADP responses in *Tpcn1/2*^−/−^ MEFs by acting as Ca^2+^-permeable channels, we generated lentiviruses for expression of TPC2-containing point mutations that affect permeability of the channel to Ca^2+^: N257A acts as a pore-dead mutant, whereas E643A has a reduced Ca^2+^ selectivity (Schieder *et al*, 2010) (Fig6A). Both mutants of TPC2 were expressed at similar levels and in the same endo-lysosomal compartments as wild-type TPC2 (Fig6B–D). While expression of wild-type TPC2 completely restored NAADP responses in *Tpcn1/2*^−/−^ MEFs, neither of the TPC2 mutants was able to rescue the response (Fig6E and F). This suggests that TPC2 must not only be a functional channel to restore NAADP action but one with a sufficient permeability to Ca^2+^.

### N-terminally truncated TPCs rescue NAADP-induced Ca^2+^ release

In the recent studies challenging TPCs as NAADP-regulated Ca^2+^-permeable channels, the *Tpcn1* and *Tpcn2* gene disruptions present in the *Tpcn1/2*^Dren^ line were proposed to potentially result in expression of truncated, dysfunctional versions of TPC1 and TPC2 (Wang *et al*, 2012; Cang *et al*, 2013). However, that they were indeed dysfunctional was not confirmed at the level of cytosolic Ca^2+^ signals, and so we generated and tested the self-same N-terminal truncated forms of mouse TPC1 or TPC2 in which only the first 69 or 49 respective amino acid residues are missing (Fig7A); it is important to note that ΔN69-TPC1 is equivalent to TPC1B, a protein predicted to be translated from a naturally occurring *Tpcn1B* isoform (Ruas *et al*, 2014) (Fig1F and G). The maximum expression level attained with either truncated form was lower than their full-length counterparts (Fig7B and C and Supplementary Fig S7), but nonetheless they were endo-lysosomal, showing a strong co-localization with LysoTracker Green (Fig7D). In spite of the lower expression, each truncated TPC remained able to rescue NAADP responsiveness, both in amplitude of Ca^2+^ signals (50–65% of that seen with their full-length equivalents) and in the number of responding cells (70–100% of transduced cells) (Fig7E–G).

These data raise doubts about whether the *Tpcn1/2*^Dren^ mice used in the previous studies (Wang *et al*, 2012; Cang *et al*, 2013, 2014) were TPC-null animals, and this may explain why preparations from pancreatic islets from these animals still retained NAADP-induced Ca^2+^ signals (Wang *et al*, 2012).

### *Tpcn1/2*^−/−^ mouse liver retains high-affinity NAADP-binding proteins

Recent studies using a radiolabelled NAADP photoaffinity probe identified putative NAADP-binding proteins in several cell preparations (Lin-Moshier *et al*, 2012; Walseth *et al*, 2012a,b) that interact with TPCs and show high-affinity specific binding to NAADP (Ruas *et al*, 2010; Walseth *et al*, 2012a). Based on their apparent molecular weights, which are lower than those predicted for TPCs and on results from transgenic mouse lines with gene trap insertions in either *Tpcn1* or *Tpcn2* genes, it was suggested that these proteins were distinct from TPCs and that an accessory NAADP-binding protein confers regulation by NAADP (Lin-Moshier *et al*, 2012; Walseth *et al*, 2012a). However, the conclusive proof that NAADP binding does not require TPC proteins demands the analysis of tissue with complete absence of both TPC1 and TPC2 proteins.

**Figure 8:**
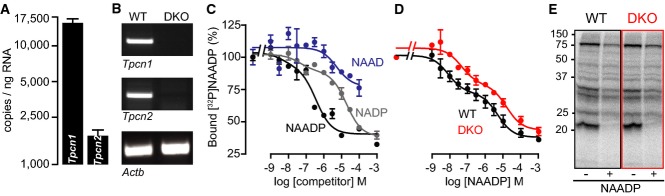
TPC proteins are not necessary for NAADP binding

We therefore compared NAADP binding in mouse liver from WT or *Tpcn1/2*^−/−^ mice, using a [^32^P]NAADP-binding assay. Liver was chosen, as we have previously shown that this tissue shows high levels of NAADP binding (Calcraft *et al*, 2009). Quantitative RT–PCR revealed that in liver from WT mice both *Tpcn1* and *Tpcn2* are expressed, albeit at different levels, with *Tpcn1* mRNA being approximately 40-fold more abundant than *Tpcn2* mRNA (Fig8A). As expected, *Tpcn1* and *Tpcn2* mRNAs were not detected in liver preparations from *Tpcn1/2*^−/−^ animals (Fig8B).

A RT–qPCR analysis of absolute levels of *Tpcn1* and *Tpcn2* transcripts in liver from WT animals. *Tpcn1*/*Tpcn2* ratio of expression corresponds to 43.9; *n *= 6.

B RT–PCR analysis of *Tpcn1* and *Tpcn2* expression in wild-type (WT) or *Tpcn1/2*^−/−^ (DKO) liver preparations. Expression of *Actb* was used as a control. Amplified cDNA regions correspond to the same exons as in Fig1D.

C, D [^32^P]NAADP-binding assay with competition by NAADP and NAADP-related dinucleotides performed with liver homogenates from WT (C, D) or DKO (D) animals. Data are expressed as values relative to total binding performed in the absence of unlabelled dinucleotide. (D) The IC_50_ values for the high-affinity binding site were WT: 13.7 ± 7.5 nM and DKO: 146.3 ± 60.5 nM (*P* > 0.09) and for the low-affinity site WT: 6.9 ± 4.1 μM and DKO: 27.4 ± 8.3 μM (*P* > 0.08); *N* (number of animals) = 5–7; *n* (number of binding reactions) = 10–14.

E Photoaffinity labelling of liver homogenates from WT or DKO animals performed with [^32^P]5N_3_-NAADP in the presence or absence of unlabelled NAADP (1 μM).

Data information: Error bars represent SEM. Source data are available online for this figure.

[^32^P]NAADP binding with unlabelled NAADP competition performed in liver homogenates from WT animals shows the characteristic binding curve revealing two populations of binding sites (Calcraft *et al*, 2009) (Fig8C and D) with higher affinity for NAADP when compared to related pyridine dinucleotides such as NADP or NAAD (Fig8C). Importantly, [^32^P]NAADP binding was retained in similar preparations from *Tpcn1/2*^−/−^ animals and showed similar IC_50_ values for both the high-affinity and low-affinity binding sites (Fig8D). Furthermore, photoaffinity labelling of NAADP-binding proteins in liver homogenates carried out using [^32^P]5N_3_-NAADP revealed no differences in the pattern of specifically labelled proteins, as assessed by competition with unlabelled NAADP (Fig8E).

Together, the data indicate that high-affinity NAADP binding does not require TPCs and support the hypothesis that an auxiliary NAADP-binding protein confers NAADP regulation.

## Discussion

In spite of compelling evidence from different groups (Morgan & Galione, 2014), recent studies have challenged the fundamental premise that TPCs are essential components of the NAADP-regulated channel, either by putting forward other target channels (Zhang *et al*, 2009; Guse, 2012) or, more recently, by suggesting that TPCs are lipid-activated Na^+^-selective channels entirely dispensable for NAADP action (Wang *et al*, 2012).

In view of such contentions, we have investigated the role of TPCs in NAADP-dependent signalling in embryonic fibroblasts from *Tpcn1/2*^−/−^ mice that we have developed (the first demonstrable TPC1/2-null system). This has allowed us to express various channels on a null background, record endogenous endo-lysosomal TPC currents, and image Ca^2+^ signals in the same cell type, permitting a direct comparison of results.

### TPCs are essential effectors of NAADP action

Our data overwhelmingly suggest that TPCs are essential for NAADP-induced Ca^2+^ signalling. We conclude this because: (i) NAADP-dependent Ca^2+^ responses were eliminated in *Tpcn1/2*^−/−^ cells whereas Ca^2+^ storage, pH_L_, and PI(3,5)P_2_ responsiveness were preserved; (ii) NAADP responses were selectively rescued by TPCs and not by another Ca^2+^-permeant endo-lysosomal channel, TRPML1 (Zong *et al*, 2009; Dong *et al*, 2010; Yamaguchi *et al*, 2011); and (iii) eradication of NAADP-regulated Ca^2+^ signalling in *Tpcn1/2*^−/−^ cells cannot be explained by incidental loss of NAADP-binding proteins since they are still present in *Tpcn1/2*^−/−^ preparations. Our data thus reinforce conclusions reached in our previous studies where NAADP responses were abrogated in cells from *Tpcn2*^−/−^ mice (Calcraft *et al*, 2009; Tugba Durlu-Kandilci *et al*, 2010).

Although clearly essential, are TPCs actually activated by NAADP? Our electrophysiological recordings suggest that they are. NAADP-evoked currents were robust in planar patch-clamp recordings of single endo-lysosomes from WT but undetectable in *Tpcn1/2*^−/−^ or *Tpcn2*^−/−^ preparations. Hence, TPC activation is relatively direct and not secondary to NAADP-induced changes in membrane potential since recordings were carried out under voltage-clamp. Under these conditions, TPC2 appears to be the predominant NAADP-activated channel; we do not currently understand why endogenous TPC1 does not contribute currents in this system (as evidenced from *Tpcn1*^−/−^ and *Tpcn2*^−/−^ preparations), even though TPC1 supports NAADP-induced Ca^2+^ release as we have shown in the rescue experiments; it is possible that TPC1-decorated endosomes are simply absent from the organelle preparation or its coupling to NAADP is less robust and lost upon purification.

Our recordings differ from those of the recent papers in several key ways: first, we successfully observed NAADP-stimulated currents in endo-lysosomal preparations, which mirrors previous work (Zhang *et al*, 2009; Pitt *et al*, 2010, 2014; Schieder *et al*, 2010; Rybalchenko *et al*, 2012; Grimm *et al*, 2014; Jha *et al*, 2014), whereas others, surprisingly, could not detect NAADP-dependent currents (irrespective of TPC expression) (Wang *et al*, 2012; Cang *et al*, 2013). Second, the scale of endo-lysosomal currents is different: our endogenous NAADP-dependent currents are in the pA range, whereas lipid-stimulated currents were in the nA range in other studies (Wang *et al*, 2012; Cang *et al*, 2013; Jha *et al*, 2014).

It is unlikely that the ability to observe NAADP-induced currents is a function of the patch-clamp technique used; others using a conventional patch-clamp technique have also been able to record NAADP-stimulated currents in endo-lysosomal preparations (Jha *et al*, 2014). However, it is possible that under some experimental conditions, necessary components of the NAADP-regulatory pathway are lost and/or inhibitory factors such as Mg^2+^ or TPC phosphorylation state (Jha *et al*, 2014) are more prevalent.

### Validity of *Tpcn* knockout mouse models

The recent conclusion that TPCs are not activated by NAADP (Wang *et al*, 2012) arose from the assumption that the *Tpcn1/2*^Dren^ mice were TPC-null, but we raise doubts as to whether their mice were true knockouts. First, no mRNA or protein expression data were presented. Second, these mice may still express functional, shorter TPC variants as we shall now discuss.

The authors' Cre-Lox strategy excised exons 1 and 2 of *Tpcn1* and exon 1 of *Tpcn2* (Wang *et al*, 2012; Cang *et al*, 2013, 2014), thereby removing the initiating ATG codon. Consequently, as the authors conceded, N-terminally truncated proteins (≥ 91% of the full-length sequence) could still be produced via initiation of translation at a downstream ATG codon (positions 70 and 50 for TPC1 and TPC2, respectively). Although these variants were dismissed as inactive channels on the basis of their PI(3,5)P_2_ insensitivity (Wang *et al*, 2012), we clearly show that these ΔN69-TPC1 or ΔN49-TPC2 proteins are functional in response to NAADP; these proteins correctly localized to endo-lysosomes (see also Ruas *et al*, 2014) and supported NAADP-induced Ca^2+^ signals in our *Tpcn1/2*^−/−^ MEFs.

Moreover, the expression of truncated TPCs can indeed occur physiologically; at least for *Tpcn1,* there is an alternative promoter downstream of exon 2 (Ruas *et al*, 2014), and mRNA for this novel shorter variant *Tpcn1B* (which gives rise to TPC1B, equivalent to ΔN69-TPC1) is present in MEFs from WT mice (but not in MEFs from our *Tpcn1/2*^−/−^ mice).

The presence of either (or both) of these shorter functional TPC proteins in the *Tpcn1/2*^Dren^ mice (Wang *et al*, 2012; Cang *et al*, 2013, 2014) would mean that they are not *bona fide Tpcn1/2* double knockouts; these studies could potentially be misleading in their claims that TPCs are not essential for NAADP-evoked Ca^2+^ signals.

### TPCs as Ca^2+^-permeable channels

Another recent controversy has been whether TPCs are Ca^2+^-permeable channels (Wang *et al*, 2012; Cang *et al*, 2013), despite different groups describing TPCs as permeant to Ca^2+^, or to Ca^2+^ surrogates, in lipid bilayers (Pitt *et al*, 2010, 2014; Rybalchenko *et al*, 2012), single-organelle planar patch-clamp (Schieder *et al*, 2010), or TPCs targeted to the plasma membrane (Brailoiu *et al*, 2010; Yamaguchi *et al*, 2011; Jha *et al*, 2014). By necessity, such experiments relied on TPC over-expression, but it is unclear whether heterologous expression truly replicates the properties of endogenous TPCs, a known complication in the TRP or Orai channel fields where different expression levels influence channel regulation, oligomerization states, and, crucially, ion selectivity (Putney, 2004; Thompson & Shuttleworth, 2013).

We conclude that TPCs are indeed Ca^2+^ permeant from multiple lines of evidence. First, Ca^2+^ fluxes through TPCs are critical for supporting NAADP-induced Ca^2+^ release because mutant TPC2 channels with a reduced or negligible Ca^2+^ permeability (Schieder *et al*, 2010) fail to rescue NAADP responses in *Tpcn1/2*^−/−^ cells. Importantly, the E643A mutant is a proven active cation channel—albeit with an altered selectivity filter (Schieder *et al*, 2010)—providing evidence that cation fluxes *per se* are not enough to support NAADP responses and that a sufficient Ca^2+^ flux is required. This is further underscored by the lack of rescue by another cation channel, TRPML1.

More direct evidence for Ca^2+^ permeability came from endo-lysosomal patch-clamp studies. Fortuitously, the endogenous endo-lysosomal NAADP-stimulated currents in MEFs are larger than those of endogenous currents in non-transfected HEK293 cells used previously (Schieder *et al*, 2010), allowing us to directly address whether endogenous TPCs are permeant to Ca^2+^. We recorded whole-lysosome native currents with Ca^2+^ and K^+^ in the lumen: in a mixed solution protocol, NAADP-stimulated currents exhibited a high permeability of Ca^2+^ over K^+^ with a reversal potential of +75 mV that was in excellent agreement with the equilibrium potential calculated for Ca^2+^ (*E*_Ca_ +73 mV); under bi-ionic conditions, the *P*_Ca_/*P*_K_ was quantified as ∼270. These indicate that, under these conditions, endogenous mouse TPCs are highly selective for Ca^2+^ over K^+^, in agreement with our previous results with mouse channels (Schieder *et al*, 2010; Grimm *et al*, 2014).

By contrast, the overall NAADP-stimulated TPC current is less discriminatory between Na^+^ and Ca^2+^ with a *P*_Ca_/*P*_Na_ of 0.6–0.8. Therefore, we agree that TPCs are permeable to Na^+^ (Wang *et al*, 2012; Cang *et al*, 2013, 2014; Boccaccio *et al*, 2014; Grimm *et al*, 2014; Jha *et al*, 2014; Pitt *et al*, 2014), but under our experimental conditions, we still observe a comparable Ca^2+^ flux. The simplest explanation of our data is that TPCs are Ca^2+^-permeable cation channels (and not highly Na^+^-selective), which broadly agrees with other studies showing permeability of mammalian TPCs to various cations such as K^+^, Cs^+^, Ba^2+^, Ca^2+^, Na^+^ and H^+^ (Brailoiu *et al*, 2010; Pitt *et al*, 2010, 2014; Schieder *et al*, 2010; Yamaguchi *et al*, 2011; Rybalchenko *et al*, 2012; Boccaccio *et al*, 2014; Grimm *et al*, 2014; Jha *et al*, 2014).

The alternative model for NAADP-induced Ca^2+^ release states that any stimulation of NAADP-induced Ca^2+^ release by TPCs could be an indirect consequence of TPC-mediated Na^+^ fluxes (Wang *et al*, 2012; Cang *et al*, 2013). However, such Na^+^ currents would inhibit Ca^2+^ release by depolarizing endo-lysosomes and reducing the electrochemical gradient for Ca^2+^ (Morgan & Galione, 2014). To accommodate TPCs as Na^+^-selective channels in NAADP-induced Ca^2+^ release would require a more complex circuit, for example involving voltage-gated or Na^+^-stimulated Ca^2+^-permeable channels (Morgan & Galione, 2014) for which there is currently no electrophysiological evidence. Moreover, NAADP signalling does not appear to require Na^+^ because it evokes a robust Ca^2+^ release from sea urchin egg homogenates in Na^+^-free media (Genazzani *et al*, 1997).

Taken together, we conclude that endogenous TPCs act as Ca^2+^-permeable channels stimulated by NAADP, consistent with the original model (Brailoiu *et al*, 2009; Calcraft *et al*, 2009; Zong *et al*, 2009) and that they are not Na^+^-selective counter-ion current facilitators.

### Modulation by PI(3,5)P_2_

Recent reports demonstrated that TPCs, like TRPML1, are regulated by the endo-lysosome-specific lipid, PI(3,5)P_2_ (Dong *et al*, 2010; Wang *et al*, 2012; Cang *et al*, 2013; Boccaccio *et al*, 2014; Jha *et al*, 2014; Pitt *et al*, 2014), and our data agree with this conclusion: PI(3,5)P_2_ stimulated robust Ca^2+^-permeable endo-lysosomal currents, and the lipid-stimulated currents were reduced in *Tpcn1/2*^−/−^ MEFs, consistent with a TPC-dependent component of the PI(3,5)P_2_ response. The residual PI(3,5)P_2_-stimulated current is attributable to other endogenous channels, a likely candidate being TRPML1 given the characteristic inward rectifying curve (Dong *et al*, 2010).

Therefore, PI(3,5)P_2_ activates multiple channel families such as TPCs, TRPML1 and RyR (Dong *et al*, 2010; Touchberry *et al*, 2010; Wang *et al*, 2012; Feng *et al*, 2014) consistent with its being a permissive lipid factor [analogous to PI(4,5)P_2_ in the plasma membrane (Suh & Hille, 2008)], whereas NAADP effects on endo-lysosomes appear to be uniquely dependent upon one channel family, the TPCs.

### TPCs and NAADP binding

Recent studies suggest that NAADP may not bind to TPCs directly but via a smaller molecular weight NAADP-binding protein(s) (Lin-Moshier *et al*, 2012; Walseth *et al*, 2012a,b) that co-immunoprecipitates with TPCs as part of a channel complex (Ruas *et al*, 2010; Walseth *et al*, 2012a). However, it is difficult to rule out whether NAADP binds TPCs directly, because in a previous study, single *Tpcn1* or *Tpcn2* knockout mice were used and the gene disruption strategy used to generate them meant that large portions of TPC proteins could potentially still be produced (Lin-Moshier *et al*, 2012). Therefore, the use of our *bona fide* double TPC1/2-null system has allowed us to conclude that TPCs are not required for high-affinity NAADP binding, as judged by crude homogenate binding studies or photoaffinity radiolabelling of mouse liver proteins, with the caveat that low-abundance TPCs may not be detected via photoaffinity labelling and/or if other more abundant NAADP-binding proteins (not related to its Ca^2+^-release properties) mask any TPC contribution.

In conclusion, the use of the first demonstrable TPC double-knockout mice affirms TPCs as Ca^2+^-permeable channels that are absolutely required for NAADP-stimulated Ca^2+^ signalling and supports PI(3,5)P_2_ as a non-selective modulator of endo-lysosomal channels. Expression of various channels in this TPC-null background reinforces this conclusion in demonstrating that only Ca^2+^-permeable TPCs can rescue NAADP signals. Our data contradict recent assertions that TPCs are NAADP-insensitive Na^+^-selective channels and establish TPCs as NAADP-regulated Ca^2+^-permeable channels.

## Materials and Methods

### Generation of *Tpcn1/2*^−/−^ mice

Homozygote *Tpcn1*^T159^ (mutant allele nomenclature: *Tpcn1*^tm1Dgen^) mice (Ruas *et al*, 2014) carrying a targeted disruption of exons 4 to 5 were obtained from the European Mouse Mutagenesis Archive (EMMA) and were used with homozygote *Tpcn2*^YHD437^ (mutant allele nomenclature: *Tpcn2*^Gt(YHD437)Byg^) mice (Calcraft *et al*, 2009) for dihybrid crosses to generate mice carrying knocked-out expression for both *Tpcn1* and *Tpcn2* genes. The genotyping of animals was performed on DNA extracted from ear biopsies using the following primers: *Tpcn1* (Intron 4F: CTGGCATCTTGAGGTTTGGT; Intron 5 R: GGGCTACACTCCCAAGCATA; KO cassette F: CCAGCTCATTCCTCCCACTC; WT product size: 376 bp; Mut product size: 459); *Tpcn2* (Intron 1F: CTTCGGAGCCTTCTTTCCTT; Intron 1 R: CTGTCCCTGACGAGTGGTTT; Gene trap cassette F: GTCGGGGCTGGCTTAACTATG; WT product size: 493 bp; Mut product size: 336). Reaction products were analysed by agarose gel electrophoresis. Mice with genotype corresponding to *Tpcn1*^T159^/*Tpcn2*^YHD437^ were born at the expected Mendelian proportion (8/126; 6.35%).

### Gene expression analysis

For analysis of gene expression, RNA was extracted following an RNeasy QiaRNA extraction procedure (Qiagen) with an in-column DNase I treatment. One-step RT–PCR was performed in a reaction containing extracted total RNA, SuperScript III RT/Platinum Taq High Fidelity Enzyme Mix (Invitrogen), and gene-specific primers: *Tpcn1* (F: ATTTTCCTGGTGGACTGTCG; R: CAGAGCAGCGACTTCGTAAA; product size: 606 bp); *Tpcn2* (F: GGGCTTCATCATTTTCCTGA; R: TTGTTGGAAGTCGTCAGCAG; product size: 564 bp); *Actb* (F: TGTTACCAACTGGGACGACA; R: AAGGAAGGCTGGAAAAGAGC; product size: 573 bp). Reaction products were analysed by agarose gel electrophoresis.

For RT–qPCR, cDNA was synthesized from RNA using high-capacity cDNA Reverse Transcription kit (Applied Biosystems). cDNA was subjected to qPCR using gene-specific, intron-flanking primers for *Tpcn1* (F: CTGTCCTCTGGATGGAACCT; R: TCCATGTTGAGCGTCAGTG) and *Tpcn2* (F: CCCTGGCTGTATACCGATTG; R: GTCCCAGAGCGACAGTGG) with Universal Probes (#95 for *Tpcn1* and #106 for *Tpcn2*) in a Light Cycler 480 System (Roche). cDNA copy numbers were determined against a standard curve using a custom-made double-stranded DNA fragment containing the amplicon sequences for *Tpcn1* and *Tpcn2* (GeneArt Strings, Life Technologies).

### Immunofluorescence

Cells were fixed in 4% paraformaldehyde in PBS and permeabilized/blocked with 0.1% saponin/5% goat serum in PBS (a methanol permeabilization step was included for anti-PDI labelling). Antibody incubations were performed in PBS/0.01% saponin/5% goat serum. The primary antibodies used were anti-RFP (rat monoclonal 5F8; antibodies-online.com), anti-mCherry (mouse monoclonal 1C51; Novus Biologicals), anti-Lamp1 (rat monoclonal 1D4B; DSHB), anti-TfR (mouse monoclonal H68.4; Invitrogen), anti-EEA1 (rabbit monoclonal, C45B10; Cell Signalling Technology), and anti-PDI (rabbit monoclonal, C81H6; Cell Signalling Technology). The secondary antibodies used were derived from goat serum, cross-absorbed, and conjugates of Alexa 488 (for organelle markers) or Alexa 546 (for mCherry) (Invitrogen). Cells were viewed on a Zeiss 510 META confocal microscope, in multitrack mode, using the following excitation/emission parameters (nm): Alexa 488 (488/505–530) and Alexa 546 (543/>560).

### Intracellular Ca^2+^ measurements

MEFs were loaded with the ratiometric Ca^2+^ indicator Fura 2-AM and where indicated pre-treated with pharmacological agents before addition of NAADP/AM, followed by ATP. The maximum amplitude and the mean [Ca^2+^] were calculated on a single-cell basis. Further details are given in Supplementary Materials and Methods.

### Lysosomal currents

Whole-lysosome planar patch-clamp recordings were performed in vacuolin-enlarged lysosomes from MEF^LTA^s isolated using differential centrifugation (Schieder *et al*, 2010). The planar patch-clamp technology combined with a pressure control system (Port-a-Patch, Nanion Technologies) was applied as previously described (Schieder *et al*, 2010). Currents were recorded at room temperature (21–23°C) using an EPC-10 patch-clamp amplifier and PatchMaster acquisition software (HEKA). Data were digitized at 40 kHz and filtered at 2.8 kHz. Seal resistance was 1–3 GΩ, and the mean endo-lysosomal capacitance was 0.82 ± 0.06 pF (*n* = 27). Inward currents are defined as ion movement from the endo-lysosomal lumen to cytoplasm (Bertl *et al*, 1992).

For experiments using mixed Ca^2+^/K^+^ solutions, the cytoplasmic solution contained 60 mM KF, 70 mM K-MSA (methanesulfonate), 0.2 mM Ca-MSA, and 10 mM HEPES (pH adjusted with KOH to 7.2); luminal solution was 70 mM K-MSA, 60 mM Ca-MSA, 1 mM MgCl_2_, and 10 mM HEPES (pH adjusted with MSA to 4.6). Mannitol was used to adjust osmolarity.

For experiments using mixed Ca^2+^/Na^+^ solutions, the cytoplasmic solution contained 60 mM NaF, 100 mM Na-MSA, 0.2 mM Ca-MSA, 5 mM Hepes, and 5 mM MES (pH adjusted with NaOH to 7.2). Luminal solution was 70 mM Na-MSA, 60 mM Ca-MSA, 1 mM CaCl_2_, 5 mM Hepes and 5 mM MES (pH 4.6).

For the bi-ionic experiments, the cytoplasmic solution contained 60 mM KF, 100 mM K-MSA, 5 mM Hepes, and 5 mM MES (pH 7.2 with KOH), whereas the luminal solution was 105 mM Ca-MSA, 2 mM CaCl_2_, 5 mM Hepes, and 5 mM MES (pH 4.6). For Na^+^ experiments, all K^+^ salts were replaced by their equimolar Na^+^ version.

Currents in the absence of NAADP (or phosphoinositides) were subtracted from the currents in the presence of these stimulators as previously described (Schieder *et al*, 2010). Water-soluble diC8-PIP_2_, PI(3,5)P_2_, and PI(4,5)P_2_ were from A.G. Scientific. NAADP was from Tocris Bioscience.

### Radioligand binding assays

[^32^P]NAADP was incubated with liver homogenate samples adsorbed to nitrocellulose filters and bound radionucleotide detected and quantified by phosphor imaging. Further details are given in Supplementary Materials and Methods.

### Photoaffinity labelling

Liver homogenate samples were photo-labelled with [^32^P-5N_3_]NAADP and proteins separated by SDS–PAGE. Signal from dried gels was detected by phosphor imaging. Further details are given in Supplementary Materials and Methods.

### Statistical analysis

Data are presented as mean ± SEM and analysed by Student's *t-*test or a one-way ANOVA (with Tukey–Kramer, Dunnett's, or Kruskal–Wallis post-tests) where appropriate and significance determined as *P* < 0.05. Graphs were usually annotated using the following conventions: *P* > 0.05 (ns), *P* < 0.05 (*), *P* < 0.01 (**), and *P* < 0.001 (***). The number of responding cells (Fig7) was assessed by multiple 2 × 2 contingency tables (Fisher's exact test) with the significance threshold (α) corrected to α′ using α′ = α/[2(c−1)] where c = number of columns and significance therefore only accepted when *P* < 0.00625.

## Supporting Information

Source Data for Supplementary Figure S7

Review Process File
