# Supplementary material for: Expression of Ca2+-permeable two-pore channels rescues NAADP signalling in TPC-deficient cells
Source: EMBO J. 2015 Apr 14;34(13):1743–58. doi: 10.15252/embj.201490009 (PMC4516428; doi:10.15252/embj.201490009)
Supplement: Supplementary file 1 [file embj0034-1743-sd1.pdf]

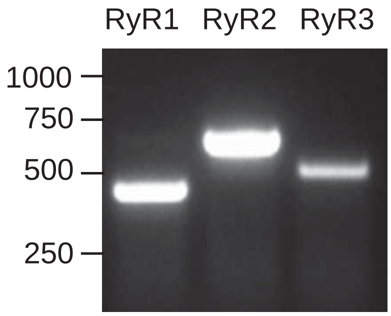

Figure S1. Expression of Ryanodine receptors in mouse brain.

RT-PCR analysis of Ryanodine Receptor expression in mouse brain, using the same primers and RT-PCR conditions as in Figure 2. In particular, the mouse brain was used as a positive control for detecting expression of RyR3, which is absent in MEFs (see Figure 2J).
