# Supplementary material for: Expression of Ca2+-permeable two-pore channels rescues NAADP signalling in TPC-deficient cells
Source: EMBO J. 2015 Apr 14;34(13):1743–58. doi: 10.15252/embj.201490009 (PMC4516428; doi:10.15252/embj.201490009)
Supplement: Supplementary file 2 [file embj0034-1743-sd2.pdf]

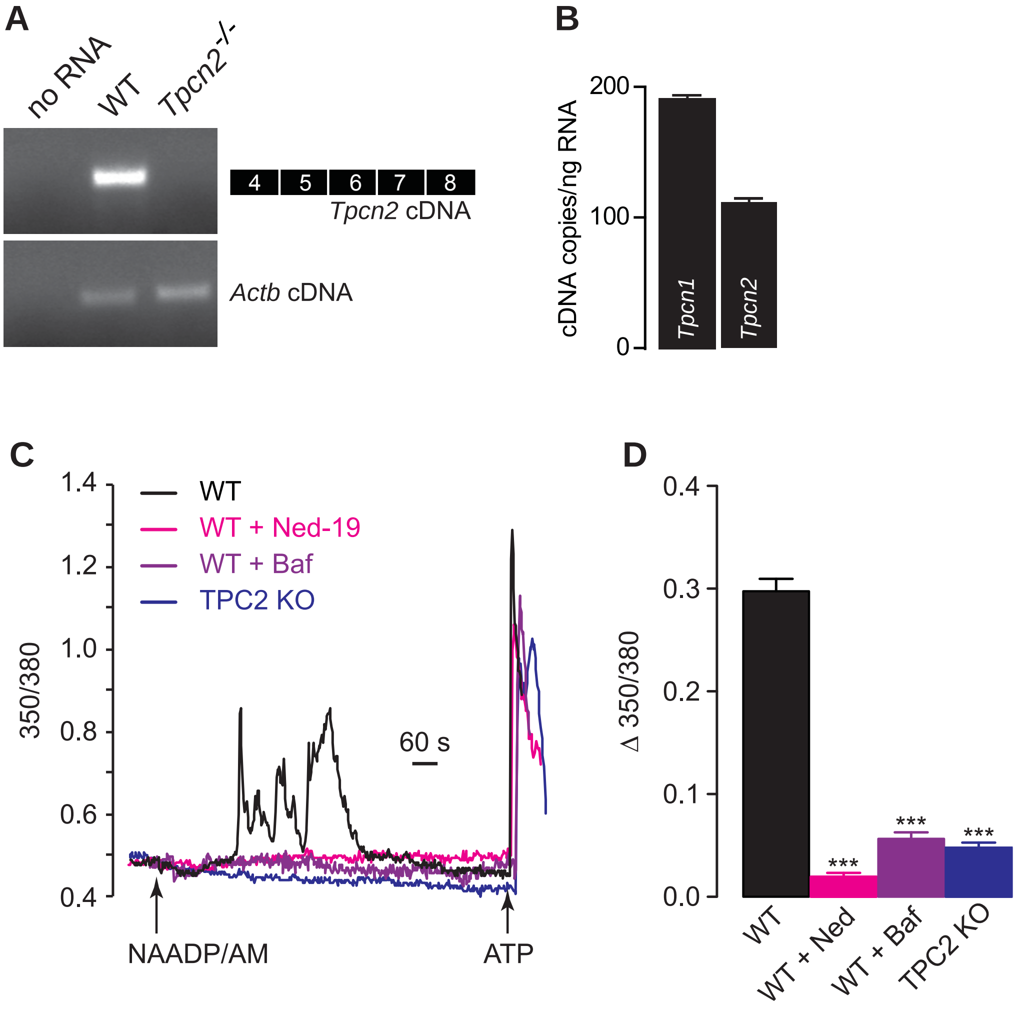

Figure S2. Macrophages from *Tpcn2*$^{-/-}$ animals fail to respond to NAADP.

A RT-PCR analysis of *Tpcn2* expression in bone marrow-derived macrophages from wild-type (WT) and *Tpcn2*$^{-/-}$ animals. Amplified cDNA corresponds to the exonic sequences shown in black. Expression of *Actb* was used as a control. Primers used for one-step RT-PCR were the same as in Figure 1.

B RT-qPCR analysis of absolute levels of *Tpcn1* and *Tpcn2* transcripts in WT macrophages. *Tpcn1*/*Tpcn2* ratio of expression correspond to 1.7; n= 6.

C Representative single-cell Ca^{2+} traces showing 350/380 ratios of fura-2 fluorescence upon addition of 5 μM extracellular NAADP/AM in wild-type (WT) and *Tpcn2*$^{-/-}$ (TPC2 KO) bone marrow-derived macrophages. NAADP/AM-induced Ca^{2+} responses were blocked in WT cells by pre-incubation with 10 μM *trans*-Ned-19 (Ned-19; 45 min) and 1 μM bafilomycin A1 (Baf; 45 min).

D Maximum Ca^{2+} changes derived from single-cell Ca^{2+} traces (as in C); n = 88–339 cells; $p < 0.0001$ (***) relative to WT cells using the ANOVA-Tukey test.
