# Supplementary material for: Expression of Ca2+-permeable two-pore channels rescues NAADP signalling in TPC-deficient cells
Source: EMBO J. 2015 Apr 14;34(13):1743–58. doi: 10.15252/embj.201490009 (PMC4516428; doi:10.15252/embj.201490009)
Supplement: Supplementary file 3 [file embj0034-1743-sd3.pdf]

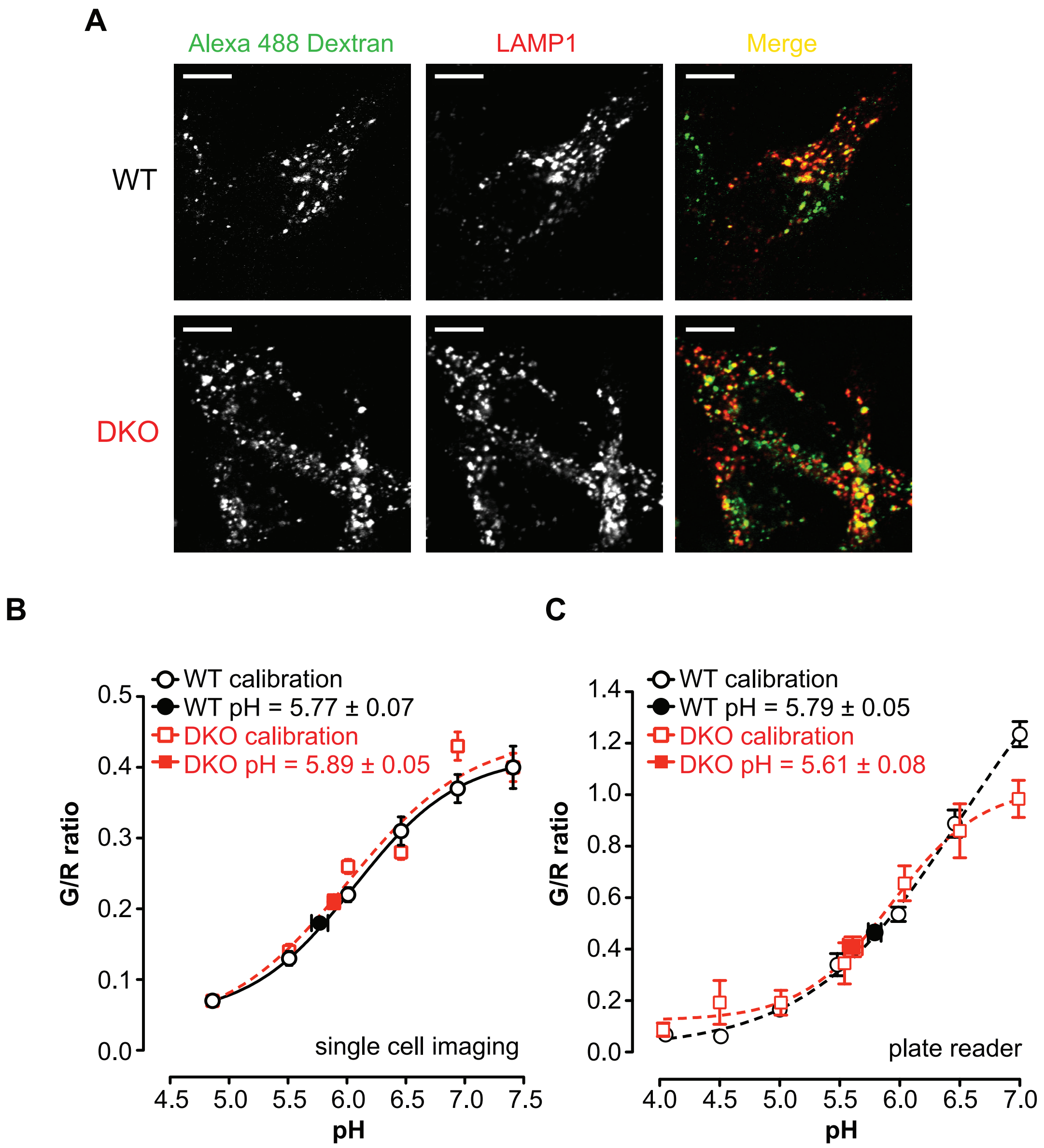

Figure S3. Determination of pH_L in endo-lysosomes of WT and DKO MEFs.

A Following loading of endo-lysosomes with a fixable Alexa 488-dextran, MEFs were fixed and immuno-labelled with an anti-LAMP1 antibody (clone 1D4B, DSHB) to confirm trafficking of the dextran to the lysosomes (as indicated by dextran co-localisation with LAMP1).

B,C MEF endo-lysosomes were loaded by endocytosis with fluorescein-dextran (pH-sensitive) and texas red-dextran (pH-insensitive) as in (A) and the fluorescence collected for single cell measurements using a confocal microscope (B) or for population analysis using a plate reader (C). Calibration curves, were obtained as described in Material and Methods. There was no significant difference between the pH_L in endo-lysosomes of wild-type (WT) and *Tpcn1/2*$^{-/-}$ (DKO) MEFs. Data are presented as the mean ± SEM: in (B) n = 105 cells (WT & DKO) and in (C) n = 16 (WT) and 12 (DKO).
