# Supplementary material for: Expression of Ca2+-permeable two-pore channels rescues NAADP signalling in TPC-deficient cells
Source: EMBO J. 2015 Apr 14;34(13):1743–58. doi: 10.15252/embj.201490009 (PMC4516428; doi:10.15252/embj.201490009)
Supplement: Supplementary file 4 [file embj0034-1743-sd4.pdf]

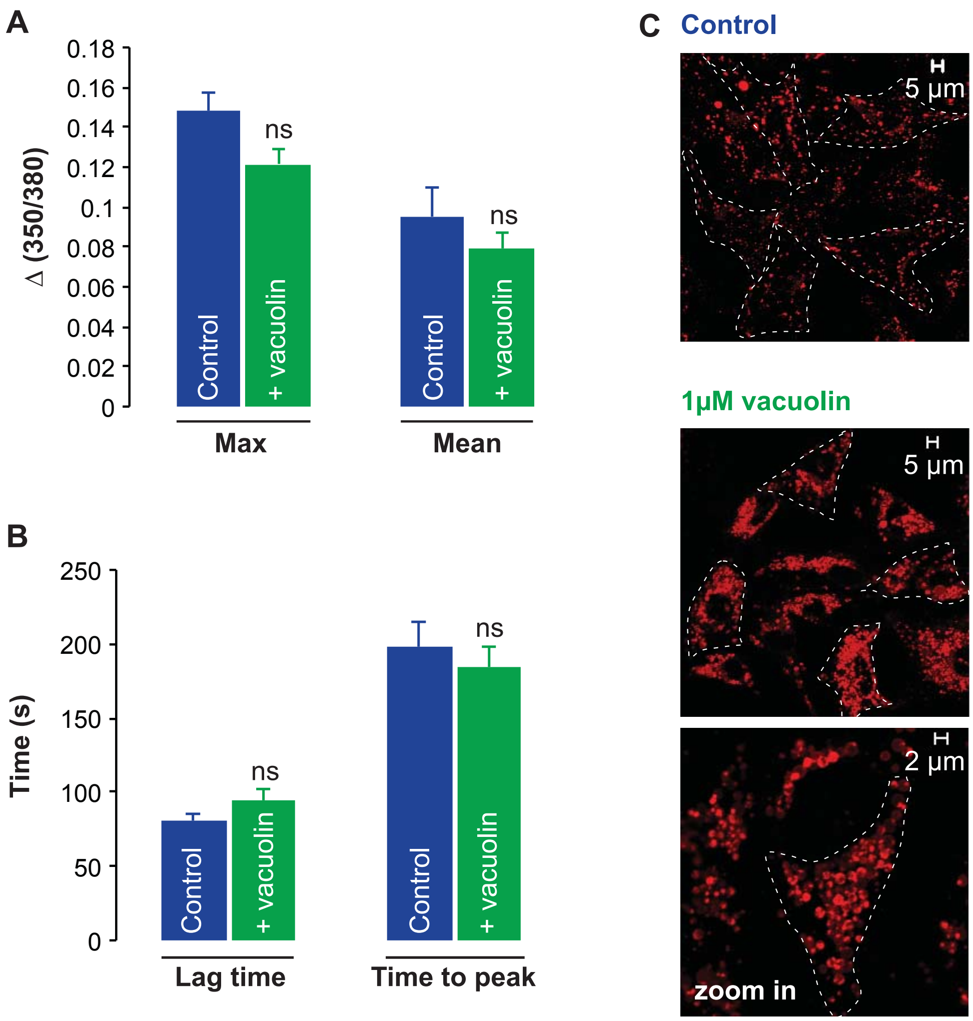

Figure S4. Vacuolin-induced enlargement of lysosomes has no significant effect on NAADP-induced Ca^{2+} release.

A-C WT MEFs were either treated with vacuolin-1 (1 µM for 4 h) or were left untreated (Control) and fura-2 Ca^{2+} signals measured after addition of 10 µM NAADP/AM in Ca^{2+}-containing medium. Vacuolin-induced enlargement of lysosomes had no significant effect on NAADP-induced Ca^{2+} release, neither in terms of amplitudes nor kinetics.

A,B (A) Maximum Ca^{2+} amplitude and the mean Ca^{2+} response over a 10-min period post-addition of NAADP/AM. (B) Lag time (delay between NAADP/AM addition and commencement of the Ca^{2+} response) and the time to peak (time period from first Ca^{2+} increase to maximum Ca^{2+} peak). n = 102-105; ns, p >0.05 (ns) relative to control using the ANOVA-Tukey test.

C Enlargement of lysosomes was confirmed by LysoTracker-Red DND99 staining (100 nM for 3 min) in WT MEFs.
