# Supplementary material for: Expression of Ca2+-permeable two-pore channels rescues NAADP signalling in TPC-deficient cells
Source: EMBO J. 2015 Apr 14;34(13):1743–58. doi: 10.15252/embj.201490009 (PMC4516428; doi:10.15252/embj.201490009)
Supplement: Supplementary file 5 [file embj0034-1743-sd5.pdf]

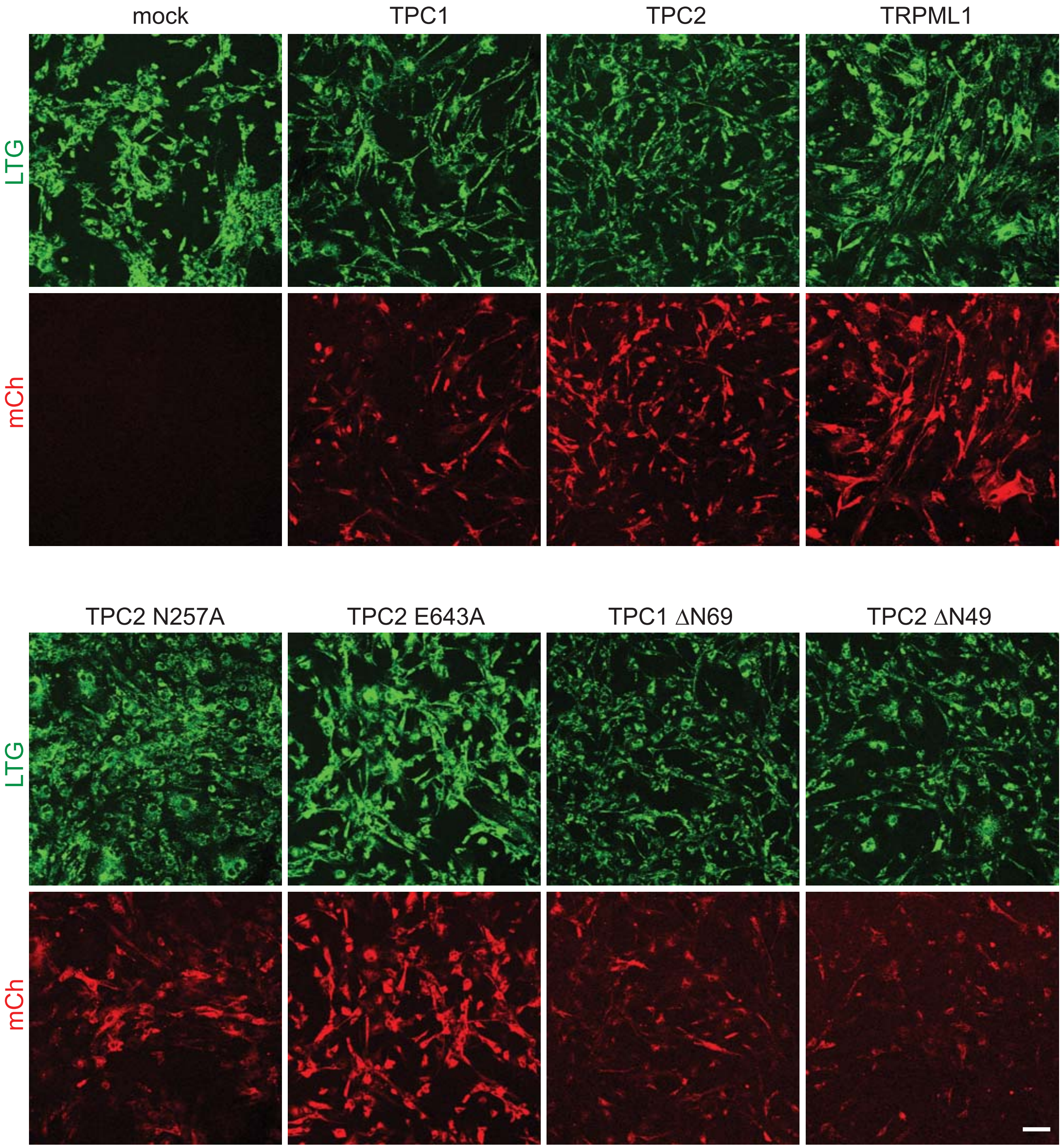

Figure S5. Imaging of C-terminal mCherry-tagged TPC proteins in MEFs.

Live-cell imaging of *Tpcn1/2*$^{-/-}$ MEF cells expressing mCherry-tagged proteins (LTG – LysoTracker Green signal; mCh – mCherry signal). Scale bar denotes 100 µm.

Each image is a copy of Figures 5C, 6C and 7C, but here, they have been subjected to unequal *post hoc* digital manipulation of the mCherry signal intensity to better visualize the signal in lower expressing cells.
