# Supplementary material for: Expression of Ca2+-permeable two-pore channels rescues NAADP signalling in TPC-deficient cells
Source: EMBO J. 2015 Apr 14;34(13):1743–58. doi: 10.15252/embj.201490009 (PMC4516428; doi:10.15252/embj.201490009)
Supplement: Supplementary file 6 [file embj0034-1743-sd6.pdf]

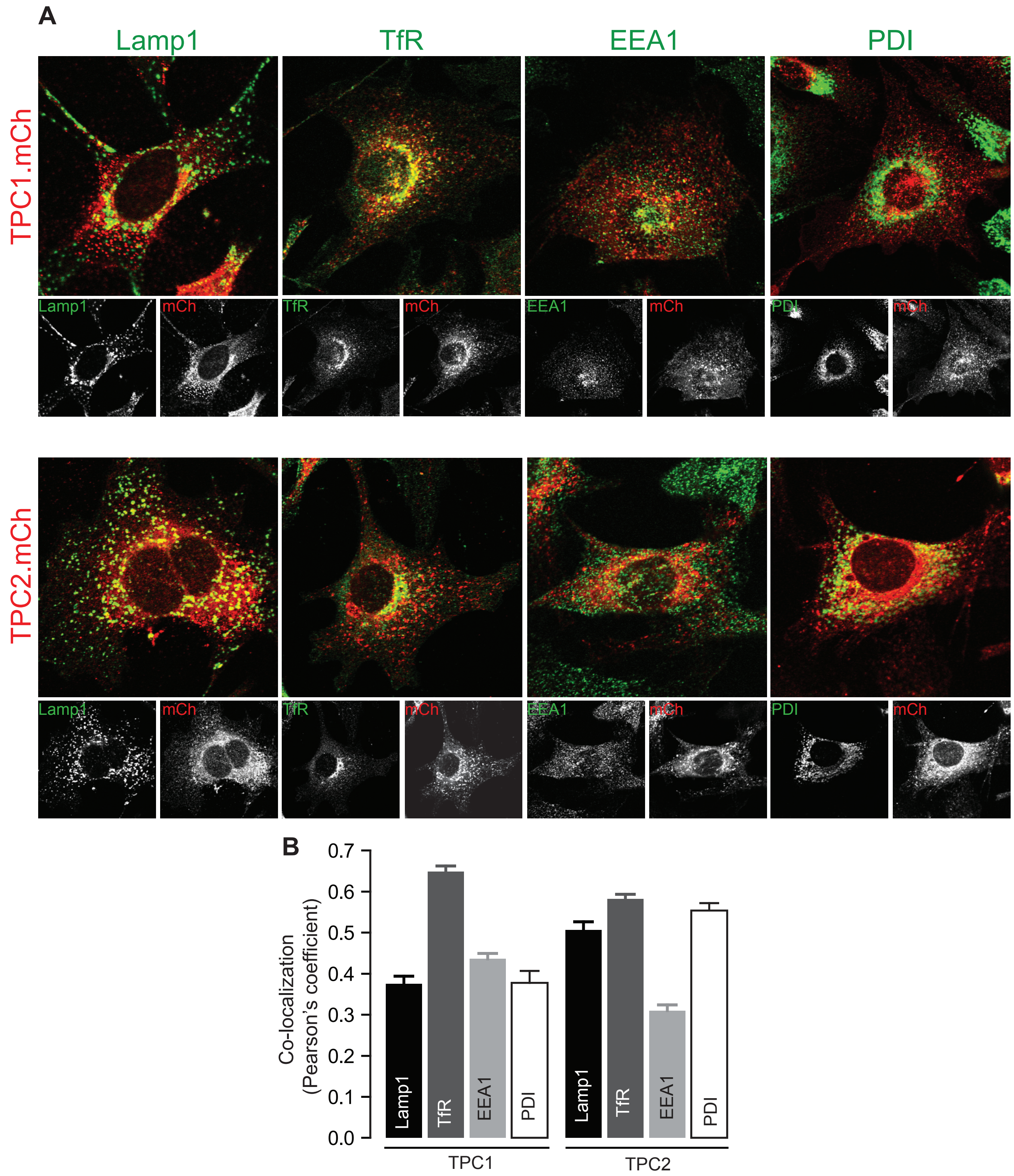

Figure S6. Co-localization of TPCs with organelle-marker proteins.

A *Tpcn1/2*$^{-/-}$ MEFs expressing mCherry-tagged TPC1 or TPC2 were immunostained with antibodies against RFP (red signal) and organelle-marker proteins (Lamp1: late endosomes/lysosomes; TfR: recycling endosomes; EEA1: early endosomes; PDI: endoplasmic reticulum; green signal). Representative images correspond to cells with a co-localization value close to the mean value for its group (B).

B Pearson's co-localization coefficient was calculated for individual cells (n = 26 - 32). Values represent mean ± SEM.

Despite the partial ER retention of TPC2.mCh in *Tpcn1/2*$^{-/-}$ MEFs, the Ca^{2+} signals evoked by NAADP in these cells retain the expected acidic Ca^{2+} store pharmacology (Fig 5G).
