# Supplementary material for: Expression of Ca2+-permeable two-pore channels rescues NAADP signalling in TPC-deficient cells
Source: EMBO J. 2015 Apr 14;34(13):1743–58. doi: 10.15252/embj.201490009 (PMC4516428; doi:10.15252/embj.201490009)
Supplement: Supplementary file 7 [file embj0034-1743-sd7.pdf]

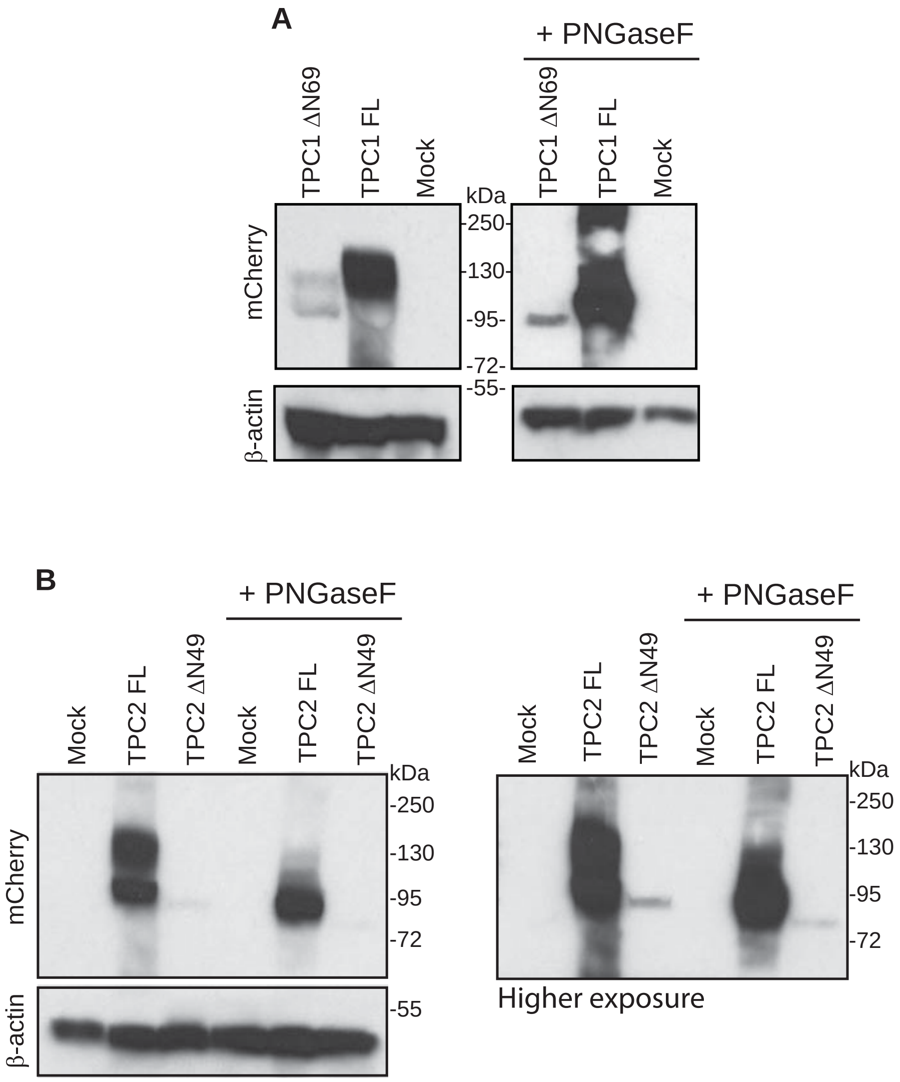

Figure S7. Glycosylation status of N-terminally truncated TPCs.

A, B Immunoblotting analysis of *Tpcn1/2*$^{-/-}$ (DKO) MEFs expressing mCherry-tagged mouse TPC1 (A) or TPC2 (B), full length (FL) and N-terminal truncations (ΔN). Blot was probed for mCherry and for β-actin as a loading control. Where indicated, lysates were subjected to PNGase F digestion as described in methods. Compared to the equivalent full-length proteins, the N-terminal truncated forms show lower levels of expression and proportionally lower levels of heavily glycosylated forms; note that only the core-glycosylated form of TPC2-ΔN49 is detected.
