# Supplementary material for: Expression of Ca2+-permeable two-pore channels rescues NAADP signalling in TPC-deficient cells
Source: EMBO J. 2015 Apr 14;34(13):1743–58. doi: 10.15252/embj.201490009 (PMC4516428; doi:10.15252/embj.201490009)
Supplement: Supplementary file 8 [file embj0034-1743-sd8.pdf]

# Selective rescue of NAADP-signalling in TPC-null cells by $Ca^{2+}$-permeable TPCs

Margarida Ruas, Lianne C. Davis, Cheng-Chang Chen, Anthony J. Morgan, Kai-Ting Chuang, Timothy F. Walseth, Christian Grimm, Clive Garnham, Trevor Powell, Nick Platt, Frances M. Platt, Martin Biel, Christian Wahl-Schott, John Parrington and Antony Galione

## SUPPLEMENTARY MATERIALS AND METHODS

### Animal husbandry

Mice were housed in individual ventilated cages at constant optimal temperature and humidity with a 12 h of light per day in the Biomedical Science Building (Oxford) and fed with standard dry pellets and water *ad libitum*. Animal use was approved by the University of Oxford's Local Ethical Review Committee and was permitted by a licence from the UK Home Office in accordance with UK law (the Animals [Scientific Procedures] Act 1986).

### Generation of mouse embryonic fibroblasts (MEFs)

MEFs were prepared from E13.5 embryos using standard protocols (Garfield, 2010) and either used as primary cells at low passage (<5 passages) or immortalized by transfection with a plasmid expressing the SV40 large T antigen (Addgene #9053; (Zalvide *et al*, 1998)) ($MEF^{LTA}$). Cells were maintained at 37ºC, 5% $CO_2$ in growth medium composed of DMEM, 10% foetal calf serum, 100 U/ml penicillin, 100 µg/ml streptomycin and 2 mM glutamine.

## Generation of macrophages

Bone marrow-derived macrophages were prepared following a standard protocol (Weischenfeldt & Porse, 2008). Briefly, bone marrow was extracted from the hind legs of 12-week old male mice and cells were plated in medium composed of RPMI, 10% foetal calf serum, 20% L929-conditioned medium, 100 U/ml penicillin, 100 µg/ml streptomycin and 2 mM glutamine. Adherent macrophage progenitors were allowed to differentiate for 7 days before analysis.

## Gene expression analysis of *RyR* and *Itpr*

For analysis of gene expression, RNA was extracted following an RNeasy QiaRNA extraction procedure (Qiagen) with an in-column DNase I treatment. One-step RT-PCR was performed in a reaction containing extracted total RNA, SuperScript III RT/ Platinum Taq High Fidelity Enzyme Mix (Invitrogen), and gene specific primers: *Ryr1* (F: GAAGGTTCTGGACAAACACGGG; R: TGCTCTTGTTGTAGAATTTGCGG; product size: 435 bp); *Ryr2* (F: GAATCAGTGAGTTACTGGGCATGG; R: CTGGTCTCTGAGTTCTCCAAAAGC; product size: 635 bp); *Ryr3* (F: CTTCGCTATCAACTTCATCCTGC; R: TCTTCTACTGGGCTAAAGTCAAGG; product size: 505 bp); *Itpr1* (F: GCTTCTACAATCCTACGACTG; R: CCTCAGGTCAGCAAAGGTGTC; product size: 737 bp); *Itpr2* (F: CCTGGCTGTGTTCATCAACCT; R: GGGCTCATCTTTCGAGGGTCG; product size: 831 bp); *Itpr3* (F: GGATCCTCATCTGCTTCTCCA; R: CGATGATCACCCCAAAGATGA; product size: 822 bp).

## Generation of lentiviruses and transduction of cells

Lentiviruses were produced by jetPEI-mediated (Source Biosciences) co-transfection of LentiX-293T cells (Clontech) with a VSV-G envelope plasmid (pMD2.VSVG),

packaging plasmid (pCMV ΔR8.91) and lentiviral vector (pLVXpuro; Clontech) carrying cDNAs (mouse *Tpcn1*: BC058951; mouse *Tpcn2*: BC141195; mouse *Mcoln1*: BC005651) with a 3' mCherry tag. Mutant cDNAs were constructed either by site-directed mutagenesis (point mutations), or by PCR using internal primers (N-truncation mutants). Transfection medium was changed after an overnight incubation and conditioned twice for 24 h. The conditioned media were pooled, filtered through a 0.45 µm filter and aliquots stored at –80ºC until use. For transduction, MEFs were seeded in coverslips, incubated overnight and medium changed to lentiviral conditioned medium containing 8 µg/ml polybrene and centrifuged at 1500 x g for 1h. Medium was changed 16 – 24 hours later and cells analysed the day after. Mock transductions were performed with conditioned medium obtained using an empty pLVXpuro vector.

### Live cell imaging

Transduced MEFs were washed twice in imaging buffer (composition in mM: 121 NaCl, 5.4 KCl, 0.8 $MgCl_2$, 1.8 $CaCl_2$, 6 $NaHCO_3$, 25 HEPES, 10 Glucose) and counterstained with 500 nM LysoTracker Green DND-26 (Invitrogen) for 5 min in imaging buffer followed by two washes. Cells were viewed on a Zeiss 510 META confocal microscope, in multitrack mode, using the following excitation/emission parameters (nm): LysoTracker (488/505-530), mCherry (543/>560).

### Immunoblotting

Transduced MEFs were lysed directly in LDS sample buffer (Pierce) and DNA disrupted by sonication. For PNGase F treatment, cells were lysed in RIPA buffer and digested overnight at room temperature following manufacturer's instructions (NEB). Proteins were resolved by SDS-PAGE (NuSep Tris-Glycine pre-cast gels), transferred to PVDF and incubated in blocking solution: Prime Block (GE Healthcare) for

chemiluminescent detection or Odyssey Block (Li-Cor Biosciences) for fluorescent detection. Membranes were incubated with a rat monoclonal anti-mCherry antibody (5F8, Chromotek) and a mouse monoclonal anti-βActin (AC-15 from Abcam or JLA20 from DSHB) as loading control. Secondary antibodies were either HRP-conjugated for chemiluminescence detection with ECL prime reagents (GE Healthcare) (Fig 5 and Fig S7) or Alexa Fluor-488 or -633 for fluorescence detection (Typhoon scanner, GE Healthcare) (Fig 6 and 7).

### Endo-lysosomal pH measurements

Primary MEFs grown in 96-well plates (plate reader assays) or on cover slips (single-cell based assays) were loaded by endocytosis with fluorescein-dextran (pH-sensitive) and Texas Red-dextran (pH-insensitive) at 0.2 mg/ml in growth media at 37 $^{o}$C for 16 h. After three washes with dextran-free media, fluorescence was collected for a population analysis using a Novostar plate reader (BMG Labtech) using excitation/emission 485/520 nm (fluorescein) and 570/620 nm (Texas Red); for single-cell based assays, fluorescence intensity was taken from confocal microscopy images (Zeiss LSM510) collected in the multitrack mode using excitation/emission 488/505-530 nm (fluorescein) and 543/>560 nm (Texas Red). Autofluorescence values from unloaded cells was subtracted from the fluorescein and Texas Red fluorescence. Fluorescein/Texas Red ratios (G/R) were calculated and pH was determined against a standard curve obtained from dextran-loaded MEFs equilibrated for at least 10 min in a high $K^+$ extracellular buffer (5 mM NaCl, 145 mM KCl, 1 mM $MgCl_2$, 1 mM $CaCl_2$, 10 mM glucose) and adjusted to a series of defined pH values in buffers (10 mM Acetate for pH 4–5; 10 mM MES for pH 5.5–6.5 and 10 mM HEPES for pH 7) containing 2 µM nigericin and 2 µM valinomycin (which collapse the membrane potential to facilitate equilibrium with external buffer). Fluorescence measurements

were acquired as above. Fluorescein- and Texas Red-dextrans (10, 000 MW) were from Invitrogen.

## Intracellular $Ca^{2+}$ measurements

MEFs were loaded with Fura 2-AM (Teflabs) at 3 µM in the presence of 0.03% Pluronic F127 (Invitrogen) at room temperature for 45 min, followed by a 15 min de-esterification. Loaded MEFs were washed with extracellular medium (mM: 121 NaCl, 5.4 KCl, 0.8 $MgCl_2$, 1.8 $CaCl_2$, 6 $NaHCO_3$, 25 HEPES, 10 Glucose) and mounted on the stage of an Olympus IX71 microscope equipped with a x40 UApo/340 objective (1.35 NA) and a 12-bit Photometrics Coolsnap HQ2 CCD camera. Cells were excited alternately by 350- and 380-nm light using a Cairn monochromator; emission data were collected at 480-540 nm using a bandpass filter. Experiments were conducted at room temperature with an image collected every 2-3 s. At the end of each run all cells responded to 100 µM ATP. Autofluorescence was determined by addition of 1 µM ionomycin with 4 mM $MnCl_2$, which quenches fura-2. Images were analysed using custom-written Magipix software (Ron Jacob, King's College London, UK) on a single-cell basis, the autofluorescence signal was subtracted and the data expressed as the maximum amplitude and the mean [$Ca^{2+}$] fura-2 ratio changes (Δ350/380). For experiments conducted in $Ca^{2+}$-free medium, MEFs were washed once with extracellular medium without added $Ca^{2+}$ and supplemented with 1 mM EGTA, followed by two washes in $Ca^{2+}$-free medium with 100 µM EGTA. Release of $Ca^{2+}$ by GPN addition was performed in $Ca^{2+}$-free medium with 100 µM EGTA. NAADP/AM was synthesised in-house (as described in (Parkesh *et al*, 2008)). The lysosomotropic agents GPN and Nigericin, were from Santa Cruz Biotechnology and Sigma, respectively. *Trans*-Ned-19 (Naylor *et al*, 2009) and Bafilomycin were from Tocris

Bioscience. Ionomycin and Cyclopiazonic Acid were from Calbiochem and 2-APB (2-aminoxydiphenylborate) was purchased from Sigma.

## Permeability ratios

Permeability ratios under bi-ionic conditions were calculated according to (Fatt & Ginsborg, 1958):

$$\frac{P_{Ca}}{P_X} = \frac{\gamma_X}{\gamma_{Ca}} \cdot \frac{[X]_i}{4[Ca]_o} \cdot exp\frac{E_{rev}F}{RT} \cdot \left[exp\left(\frac{E_{rev}F}{RT}\right) + 1\right]$$

Permeability ratios under mixed solutions were calculated using a form of the Campbell equation (Campbell *et al*, 1988):

$$\frac{P_{Ca}}{P_{Na}} = \frac{\gamma_{Na}}{4\gamma_{Ca}} \cdot \frac{\left[[Na]_i exp\left(\frac{E_{rev}F}{RT}\right) - [Na]_o\right]}{\left[[Ca]_o - [Ca]_i exp\left(\frac{2E_{rev}F}{RT}\right)\right]} \cdot \left[exp\left(\frac{E_{rev}F}{RT}\right) + 1\right]$$

Where: $P_{Ca}$ = $Ca^{2+}$ permeability; $P_{Na}$ = $Na^+$ permeability; $P_X$ = monovalent permeability; $\gamma_{Ca}$ = $Ca^{2+}$ activity coefficient (=0.52); $\gamma_X$ = monovalent ion activity coefficient (=0.75); $[Ca]_o$ = concentration of $Ca^{2+}$ in the lumen; $[Na]_o$ = concentration of $Na^+$ in the lumen; $[Na]_i$ = concentration of $Na^+$ in the cytosol; $[X]_i$ = concentration of monovalent ions in the cytosol; $E_{rev}$ = reversal potential; F,R = standard thermodynamic constants; T = temperature.

## Liver homogenates

Livers from individual animals were collected and washed in PBS before being suspended and homogenized (ULTRA-TURRAX homogenizer) in a buffer containing 20 mM Hepes, 1 mM EDTA, pH 7.2, supplemented with protease inhibitors (Complete, Roche). The homogenate was cleared from undisrupted cells and nuclei by centrifugation at 1,000 x g for 5 min at 4 ºC and aliquots were stored at -80ºC until use. Protein content was determined using a bicinchoninic acid (BCA) assay.

## Radioligand binding assays

[$^{32}$P]NAADP was synthesised in-house following two enzymatic reactions using [$^{32}$P]NAD (800 Ci/mmol, Perkin Elmer), as previously described (Morgan *et al*, 2005). Liver homogenates were adsorbed to nitrocellulose filters and incubated in binding buffer (250 mM potassium acetate, 20 mM HEPES, pH 7.2) containing increasing amounts of NAADP. After 15 min at room temperature, [$^{32}$P]NAADP was added to a final concentration of 0.2 nM and reaction was allowed to proceed for 1 h at room temperature. Filters were washed in ice-cold binding buffer and exposed to a phosphorscreen that was scanned using a Typhoon 9400 scanner (GE Healthcare). Results were fitted to a two-population of binding sites (GraphPad Prism).

## Photoaffinity labelling

Liver homogenate samples were labelled with [$^{32}$P-5$N_3$]NAADP, a partial NAADP agonist with nM affinity (Jain *et al*, 2010) as previously described (Lin-Moshier *et al*, 2012; Walseth *et al*, 2012), and proteins separated by SDS-PAGE. Dried gels were exposed to a phosphorscreen and scanned using a Cyclone storage phosphor system (Packard Instruments).

## SUPPLEMENTARY REFERENCES


Calcraft PJ, Ruas M, Pan Z, Cheng X, Arredouani A, Hao X, Tang J, Rietdorf K, Teboul L, Chuang K-T, Lin P, Xiao R, Wang C, Zhu Y, Lin Y, Wyatt CN, Parrington J, Ma J, Evans AM, Galione A, et al (2009) NAADP mobilizes calcium from acidic organelles through two-pore channels. *Nature* **459:** 596–600

Campbell DL, Giles WR, Hume JR, Noble D & Shibata EF (1988) Reversal potential of the calcium current in bull-frog atrial myocytes. *J. Physiol.* **403:** 267–286

Fatt P & Ginsborg BL (1958) The ionic requirements for the production of action potentials in crustacean muscle fibres. *J. Physiol.* **142:** 516–543

Garfield AS (2010) Derivation of Primary Mouse Embryonic Fibroblast (PMEF) Cultures. In *Mouse Cell Culture*, Ward A & Tosh D (eds) pp 19–27.

Jain P, Slama JT, Perez-Haddock LA & Walseth TF (2010) Nicotinic Acid Adenine Dinucleotide Phosphate Analogs Containing Substituted Nicotinic Acid: Effect of Modification on $Ca^{2+}$ Release. *J. Med. Chem.* **53:** 7599–7612

Morgan A, Churchill G, Masgrau R, Ruas M, Davis L, Billington R, Patel S, Yamasaki M, Thomas J, Genazzani A & Galione A (2005) Methods in Cyclic ADP-Ribose and NAADP Research. In *Calcium Signaling, Second Edition*, Putney J (ed) pp 265–334. CRC Press.

Lin-Moshier Y, Walseth TF, Churamani D, Davidson SM, Slama JT, Hooper R, Brailoiu E, Patel S & Marchant JS (2012) Photoaffinity Labeling of Nicotinic Acid Adenine Dinucleotide Phosphate (NAADP) Targets in Mammalian Cells. *J. Biol. Chem.* **287:** 2296–2307

Naylor E, Arredouani A, Vasudevan SR, Lewis AM, Parkesh R, Mizote A, Rosen D, Thomas JM, Izumi M, Ganesan A, Galione A & Churchill GC (2009) Identification of a chemical probe for NAADP by virtual screening. *Nat Chem Biol* **5:** 220–226

Parkesh R, Lewis AM, Aley PK, Arredouani A, Rossi S, Tavares R, Vasudevan SR, Rosen D, Galione A, Dowden J & Churchill GC (2008) Cell-permeant NAADP: A novel chemical tool enabling the study of $Ca^{2+}$ signalling in intact cells. *Cell Calcium* **43:** 531–538

Ruas M, Chuang K-T, Davis LC, Al-Douri A, Tynan PW, Tunn R, Teboul L, Galione A & Parrington J (2014) TPC1 has two variant isoforms and their removal has different effects on endo-lysosomal functions compared to loss of TPC2. *Mol. Cell. Biol.*

Walseth TF, Lin-Moshier Y, Jain P, Ruas M, Parrington J, Galione A, Marchant JS & Slama JT (2012) Photoaffinity Labeling of High Affinity Nicotinic Acid Adenine Dinucleotide Phosphate (NAADP)-Binding Proteins in Sea Urchin Egg. *J. Biol. Chem.* **287:** 2308–2315

Weischenfeldt J & Porse B (2008) Bone Marrow-Derived Macrophages (BMM): Isolation and Applications. *Cold Spring Harb. Protoc.* **2008:** pdb.prot5080

Zalvide J, Stubdal H & DeCaprio JA (1998) The J Domain of Simian Virus 40 Large T Antigen Is Required To Functionally Inactivate RB Family Proteins. *Mol. Cell. Biol.* **18:** 1408–1415
