# Supplementary figures and images for: Expression of Ca2+-permeable two-pore channels rescues NAADP signalling in TPC-deficient cells
Source: EMBO J. 2015 Apr 14;34(13):1743–58. doi: 10.15252/embj.201490009 (PMC4516428; doi:10.15252/embj.201490009)

**Figure S1**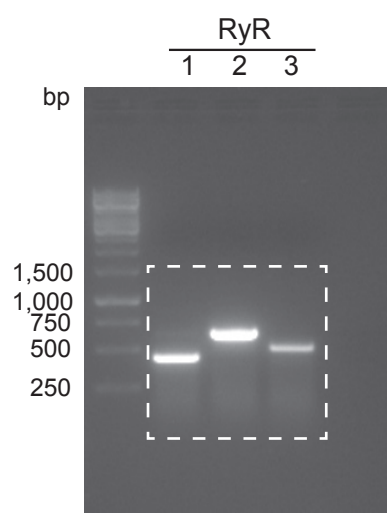

Supplement: Supplementary file 9 [file embj0034-1743-sd9.pdf]

Figure S2A

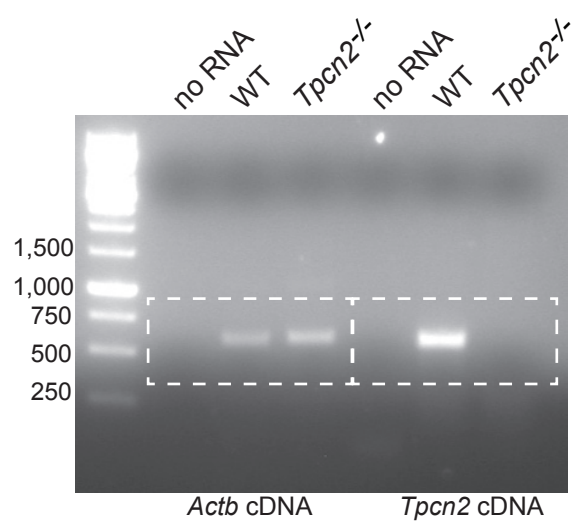

Supplement: Supplementary file 10 [file embj0034-1743-sd10.pdf]

Figure S7A

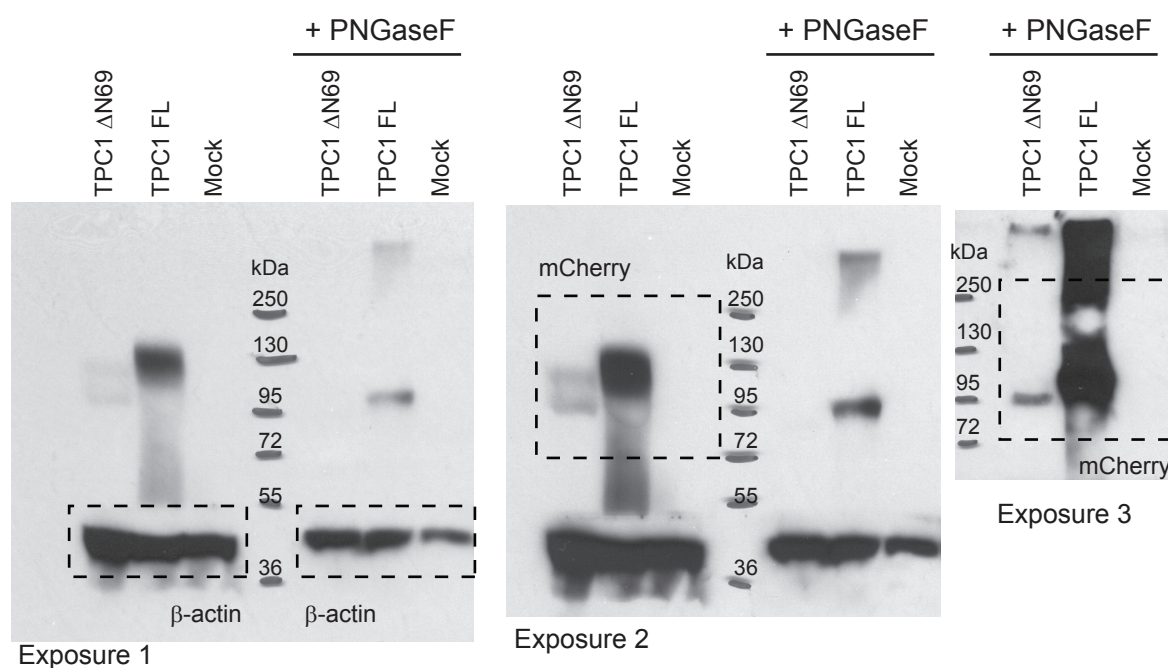

Figure S7B

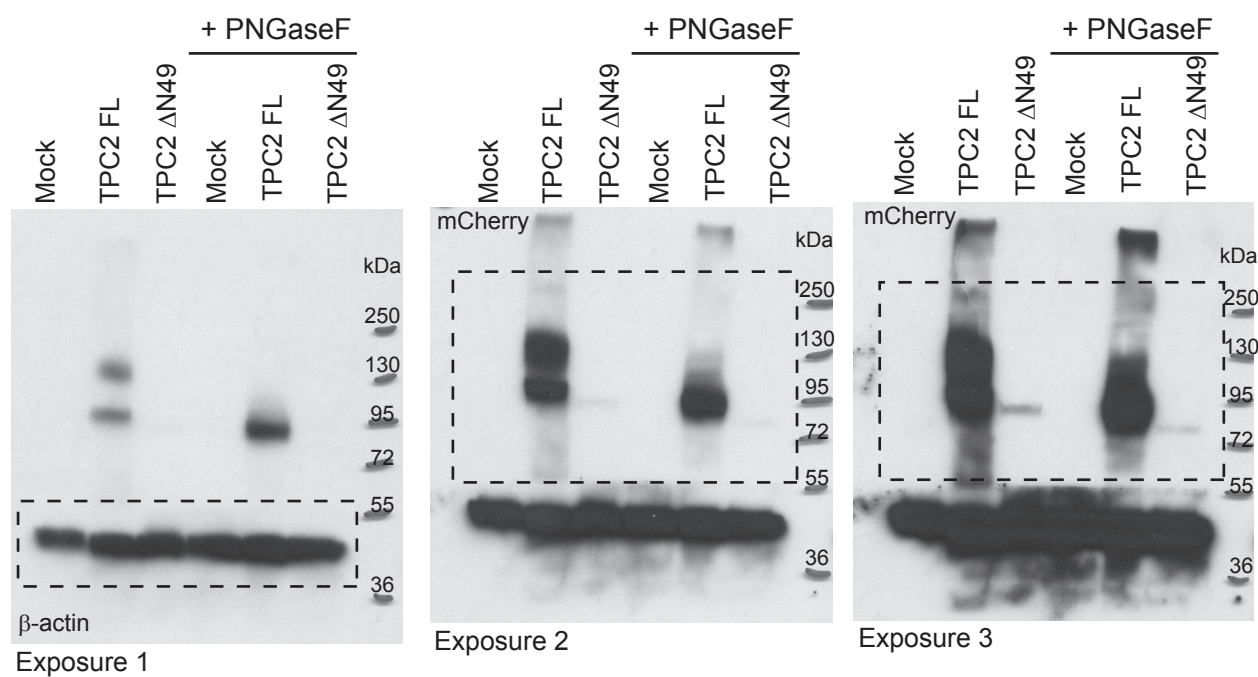

Supplement: Supplementary file 11 [file embj0034-1743-sd11.pdf]

Figure 1B

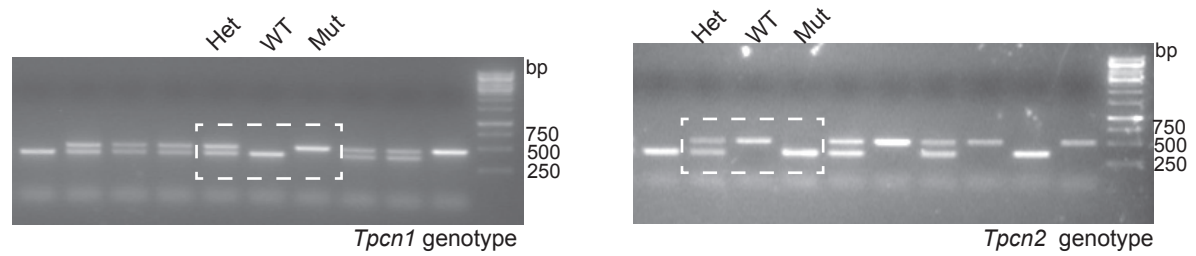

Figure 1D

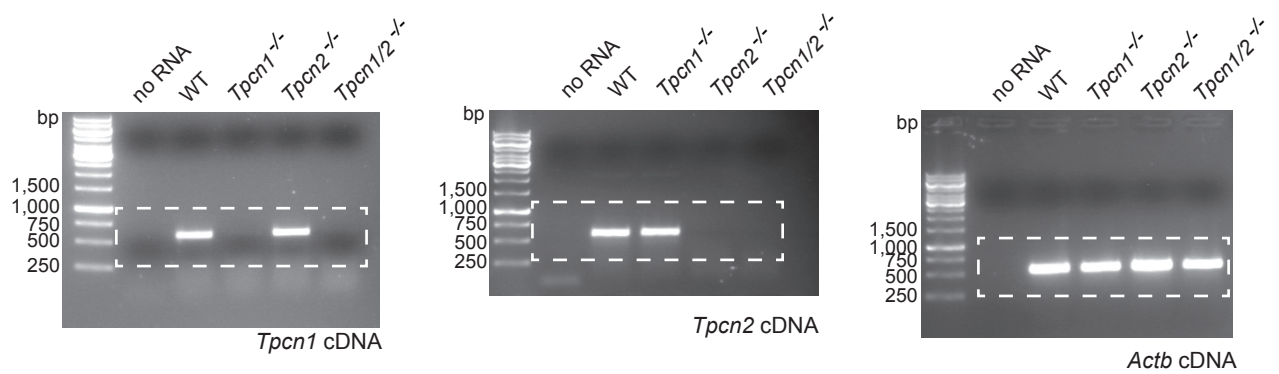

Figure 1F

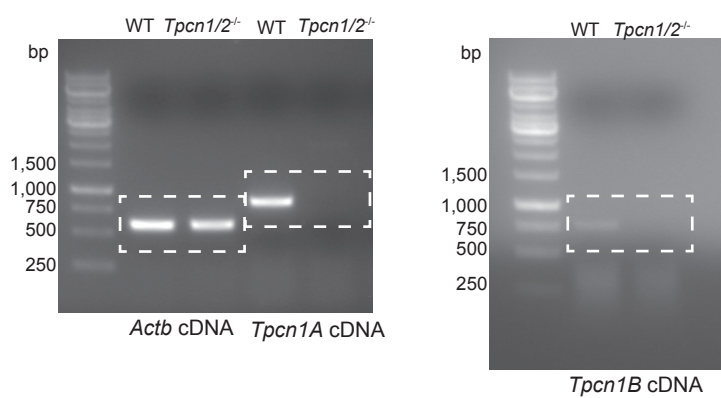

Supplement: Supplementary file 13 [file embj0034-1743-sd13.pdf]

Figure 2I

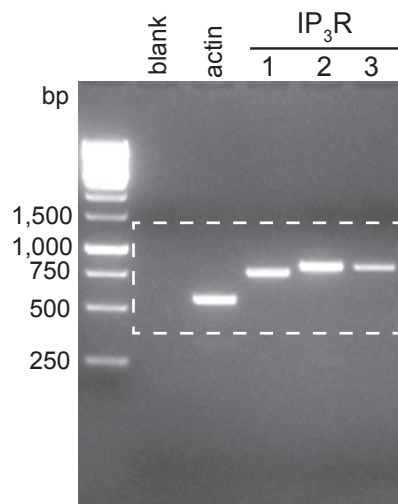

Figure 2J

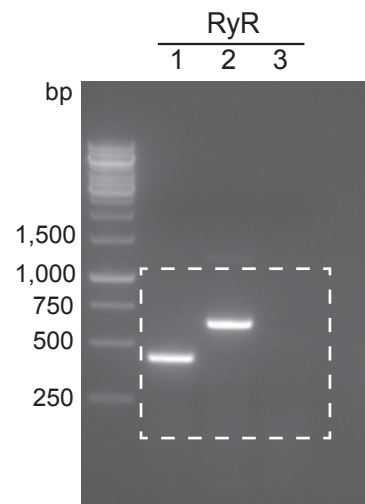

Supplement: Supplementary file 14 [file embj0034-1743-sd14.pdf]

Figure 5A

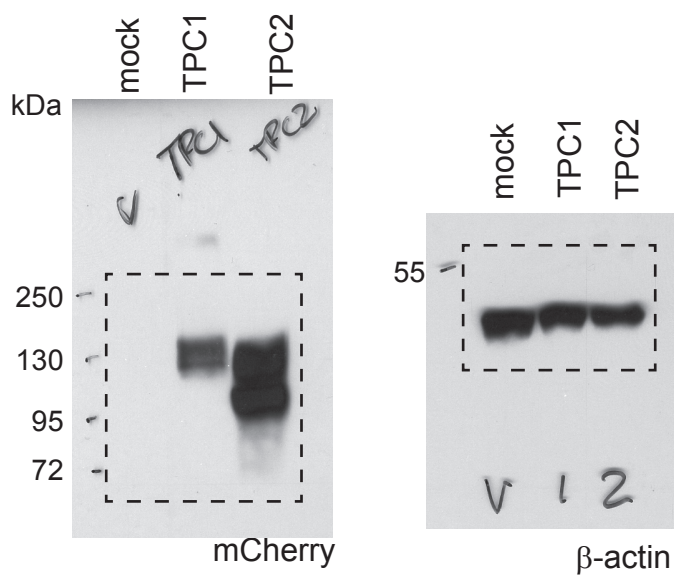

Figure 5B

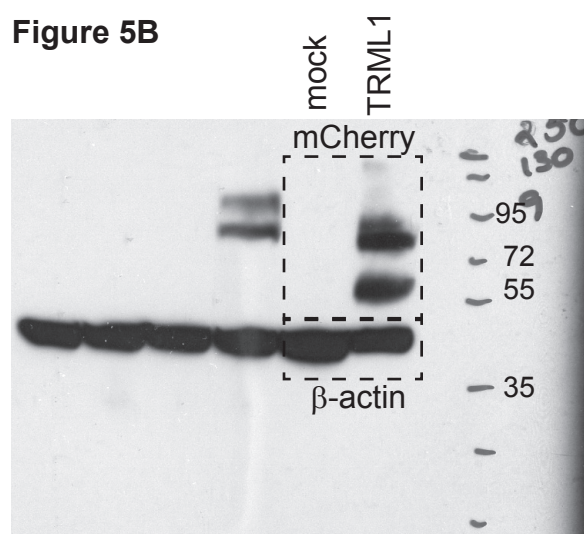

Supplement: Supplementary file 15 [file embj0034-1743-sd15.pdf]

Figure 6B

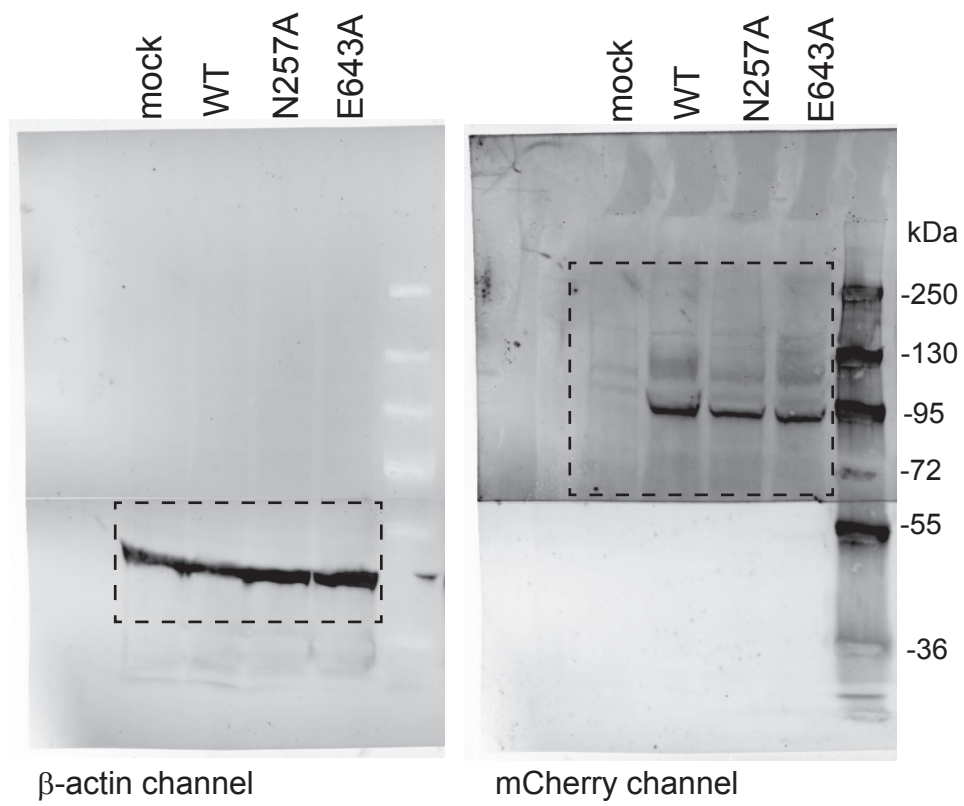

Supplement: Supplementary file 16 [file embj0034-1743-sd16.pdf]

Figure 7B

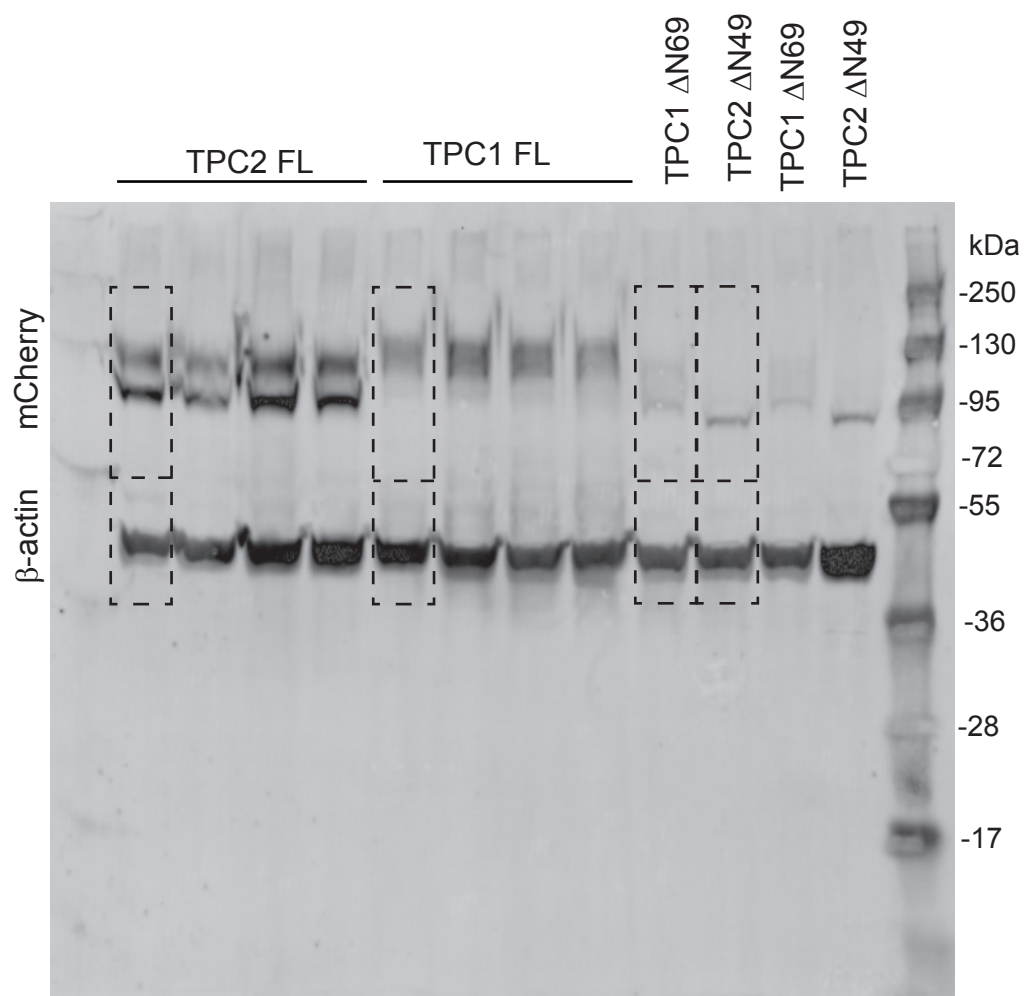

$\beta$ -actin & mCherry channels

Supplement: Supplementary file 17 [file embj0034-1743-sd17.pdf]

Figure 8B

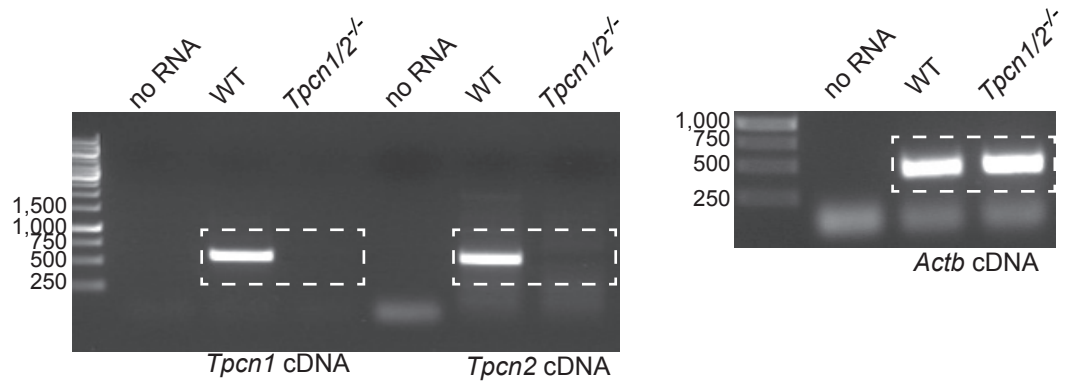

Supplement: Supplementary file 18 [file embj0034-1743-sd18.pdf]
