# Supplementary material for: Expression of Ca2+-permeable two-pore channels rescues NAADP signalling in TPC-deficient cells
Source: EMBO J. 2015 Apr 14;34(13):1743–58. doi: 10.15252/embj.201490009 (PMC4516428; doi:10.15252/embj.201490009)
Supplement: Supplementary file 12 [file embj0034-1743-sd12.pdf]

THE EMBO JOURNAL

Manuscript EMBO-2014-90009

Expression of Ca2+-permeable Two-Pore Channels (TPC) rescues NAADP-signalling in TPC-deficient cells

Margarida Ruas, Lianne C Davis, Chen Cheng-Chang, Anthony J Morgan, Kai-Ting Chuang, Timothy F Walseth, Christian Grimm, Clive Garnham, Trevor Powell, Nick Platt, Frances M Platt, Martin Biel, Christian Wahl-Schott, John Parrington, Antony Galione

Corresponding authors: Margarida Ruas, Antony Galione, John Parrington, University of Oxford

Review timeline:

Submission date:	10 September 2014
Editorial Decision:	14 October 2014
Revision received:	12 February 2015
Editorial Decision:	03 March 2015
Revision received:	06 March 2015
Accepted:	11 March 2015

Editor: Anne Nielsen

Transaction Report:

(Note: With the exception of the correction of typographical or spelling errors that could be a source of ambiguity, letters and reports are not edited. The original formatting of letters and referee reports may not be reflected in this compilation.)

1st Editorial Decision 14 October 2014

Thank you for submitting your manuscript for consideration by the EMBO Journal. It has now been seen by two referees whose comments are shown below. In addition, we have consulted with an additional expert advisor who has seen both the reports and the manuscript.

As you will see from the reports, both referees express interest in the findings reported in your manuscript and highlight the value of reconfirming the physiological role for TPC1 and TPC2 in lysosomal Ca2+ release. However, at the same time the referees do outline a number of major and minor concerns that will have to be addressed before they can support publication of a revised manuscript. More specifically, you will need to include additional experimental data to further address the ion selectivity of the TPCs (ref#1) as well as the difference in experimental settings used for comparison to previous studies (ref#2). Regarding the other comments from ref #2, it would enhance readability of the study to moderately shorten the discussion, but this is not a direct requirement from our side. The same thing goes for the final point raised by this referee, where I would rather recommend that you keep the data on NAADP binding in the manuscript.

In addition to these concerns from the referees, our expert advisor raised the following points:

'The Ca2+ measurements are solid with one exception. In all traces the NAADP-evoked signal has a sustained elevated response. The question arising is whether this response is mediated by Ca2+ influx. This can easily be tested by measuring the response to NAADP in the absence of external

© European Molecular Biology Organization

Ca2+. If it is, then the peak and extent of NAADP-evoked Ca2+ release need to be re-evaluated in wild-type, the various knockout and re-expressed conditions by measuring the release in the absence of external Ca2+.

I am more concerned about the electrophysiology, in particular the Ca2+ selectivity of the current. It is commendable that the authors measured the native NAADP-activated current and show it to be Ca2+ selective. However, in doing so they use an artificial reconstitution system that may affect channel properties. When measured in lysosomes expressing TPC1 or TPC2 (the native membranes) the current does not show Ca2+ selectivity. I would have liked the authors to have at least expressed TPC1 and TPC2 in their fibroblasts (or COS cells as was done originally) and used their recording system to show that at least one of the channels is indeed Ca2+ selective relative to K+.'

Given the referees' positive recommendations, I would like to invite you to submit a revised version of the manuscript, addressing the comments of both reviewers and advisor. I should add that it is EMBO Journal policy to allow only a single round of revision, and acceptance of your manuscript will therefore depend on the completeness of your responses in this revised version.

When preparing your letter of response to the referees' comments, please bear in mind that this will form part of the Review Process File, and will therefore be available online to the community. For more details on our Transparent Editorial Process, please visit our website: http://emboj.embopress.org/about#Transparent_Process

We generally allow three months as standard revision time. As a matter of policy, competing manuscripts published during this period will not negatively impact on our assessment of the conceptual advance presented by your study. However, we request that you contact the editor as soon as possible upon publication of any related work, to discuss how to proceed. Should you foresee a problem in meeting this three-month deadline, please let us know in advance and we may be able to grant an extension.

Thank you for the opportunity to consider your work for publication. I look forward to your revision.

REFEREE REPORTS

Referee #1:

For the last years there has been a debate as to whether the endosomal/lysosomal TPC channels mediate NAADP-triggered Ca efflux from acidic subcellular compartments. Whilst several groups found strong evidence for this notion by several methods, both the Ca selectivity and the responsiveness to NAADP have been challenged by other labs that used genetically modified cells claimed to be deficient of functional TPC1 and TPC2. Ruas et al now use newly generated knockout mice demonstrably lacking both TPCs. With cells from these as control, the authors reconfirm that TPCs are required for NAADP-induced lysosomal Ca release (live-cell Ca imaging) and for NAADP-induced endosomal/lysosomal Ca currents (planar patch-clamp of vacuolin-enlarged organelles). Importantly, they show that re-expression of TPCs (but not of transport deficient mutants of TPC2) in TPC1/2 double knockout reconstitutes NAADP-induced Ca release - even those truncated versions of TPC1 and 2 that are likely expressed in the mice previously claimed to be knock-out. Finally the authors show by a radioactive binding assay that TPCs themselves are not the NAADP receptors.

Overall this is a timely and important study that convincingly reconfirms the proposed function of TPCs as NAADP-triggered Ca release channels and which explains partly some of the reasons for the debate over the last years (previous knockout mice not really being knock-out). The study is clearly described and has been performed with the necessary controls.

Several points should be addressed before publication:
Ion selectivity of TPC: The authors tested the Na/Ca permeability ratio only by substituting 'cytosolic' K by Na, but did not test Na on the luminal side. However, Xu and coworkers postulate that lysosomal exit of Na through TPC2 is important, and luminal Na concentration may be higher

than in the cytosol (even though the determination of lysosomal Na by Xu et al. is flawed). It is conceivable that TPCs are more Na- and less Ca-permeable with Na in the inside (or both sides) of the vesicle. This should be checked electrophysiologically.

Minor points:
- pH measurements were claimed to cover "the entire endo-lysosomal system" (p.7). Most likely the authors measured lysosomal pH, but that is hard to judge because there is no description as to how they did this (loading, chase time, calibration) in the methods. These experimental details should be given.
- Fig.5C: TPC1 only partially co-localizes with LysoTracker, indicating it's early-endosomal. Please comfirm by colocalization with marker, such as EEA1. And please colocalize TPC2 with lamp1, which is more specific and preferable to LysoTracker.
- P4: calling the other groups interrelated seems a bit polemical.
- P4: last sentence: already here mention briefly why it's questionable that the previous mice are indeed knockout.
- P5, first line of results: T159, not T157
- P6 and suppl fig S1: do macrophages have only TPC2? Why not even tested for TPC1 in RTPCR? Replace 'knockdown' on page 6 by 'knockout'.
- P.17: controversy has (space missing)
- Fig.7C: pictures too dark, hard to see anything; in general, immunofluorescence pictures are too small.
- Legend Fig 1C: better "highlighted with blue bar" (was not easy to see what was meant)
- Legend Fig 2A,B: $p>0.05$ (ns) relative to control: not clear what it relates to and what it means.
- Possible differences between planar patch clamp used here and by some of the authors before and 'could be conventional' patch-clamp by the "opposing labs" could be discussed.
- Please discuss in more detail the recent paper by Jha et al. , in particular whether a difference in Mg concentration can explain the difference between the present work and those studies where no effect of NAADP on TPCs could be found.
- Could the authors perform Western blots to additionally confirm the present KO mouse lines?
- Western in Fig. 7B: I am puzzled by the large apparent size difference between TPC1 FL and TPC1 deltaN69. Further, please include a non- or mock-transfected control and run the samples on the same gel (the present one is cut and put together).
- Ca-signal in Fig. 7E: the increase of calcium concentration with the TPC1 deltaN69 is much delayed. Is this typical? Do you think it results from Ca-induced Ca-release from ER stores?

Referee #2:

NAADP induced Ca2+ release from lysosomal stores represents an important lysosomal signalling pathway. Two-pore channel proteins TPC1 and TPC2 localized at the intracellular endosomes and lysosomes (‚endo-lysosomes') were proposed to be important players in NAADP-triggered endolysosomal Ca2+ release. However, conflicting findings were reported concerning NAADP-sensitivity (and ion selectivity) of TPC channels. Importantly, a recent publication by Wang et al. in Cell reported that TPC channels are insensitive to NAADP and that TPC-mediated currents are selective for Na+, challenging the role of TPC channels as NAADP-regulated Ca2+-permeable ion channels. Here, Ruas et al., taking advantage of a TPC1/2 double knock-out (DKO) mouse line, have characterized TPC1/2 channel properties in endo-lysosomes, and come to very different conclusions. Based on their results Ruas et al. propose that TPC channels are Ca2+-permeable ion channels indispensible for NAADP signalling.

Ruas et al. tested the ability of DKO mouse embryonic fibroblasts (MEFs) to respond to NAADP. The data demonstrates that DKO MEF showed no Ca2+ response to NAADP. Measurements of TPC channel activity are technically very challenging and mostly rely on isolated vacuolin-1 swollen lysosomes, a procedure with many pitfalls and experimental artefacts. Importantly, Ruas et al. could rescue NAADP-induced Ca2+ release in DKO MEFs by lentivirus-based reexpression of mCherry tagged TPC1 and TPC2, whereas expression of TPC pore mutants failed to rescue NAADP-induced Ca2+ release. Also, the authors carried out planar-patch clamp experiments on swollen endo-lysosomes to investigate NAADP-sensitivity and ion selectivity. The data indicates that NAADP-stimulated currents displayed a rank order of selectivity of Na+ {greater than or equal to} Ca2+ >> K+. These results demonstrate that in the absence of Na+, Ca2+ is the major cation

permeating TPC channels.
In a final chapter Ruas et al. address the possibility that NAADP does not directly bind to TPC channels, but binds to an accessory protein. Based on NAADP binding and affinity labeling experiments it was concluded that NAADP does not bind directly to TPC channels and that an auxiliary NAADP-binding protein confers NAADP regulation.

Specific comments:
In Discussion Ruas et al. are mainly concerned with studies of competitors who challenged the view that TPC channels are NAADP-regulated Ca2+ premeable channels. The Discussion section is as along as the results section and should significantly be shortened. It reiterates many points that were already made in Results and need not be repeated. It suffices to state the differences, e.g. the previous mice were not null for TPC, whereas the present mice were definitely null. Furthermore, it should be emphasized that besides differing experimental conditions (overexpression in tissue culture cells) different preparations were used. Assuming that the NAADP binding protein is an accessory protein, it may not be expressed in all cells alike, e.g. HEK293, COS-1, macrophages, and MEFs. This point may need more emphasis than others, which should be discussed in a much more concise manner. In this context the authors should also comment on the fact that they measured currents in the range of 10 - 20 pA, whereas the competitors reported currents in the 1000 - 2000 pA range. This difference may have quite an impact on the interpretation of some of the electrophysiological data.

Fig. 4: It would be nice to show one experiment in the absence of Ca2+ in a Na+ only configuration similar to the conditions of Wang et al. (2012). Also, it would be helpful to include a current trace, where PI(3,5)P2 and NAADP were added together.

Fig. 8: NAADP-binding studies
This part of the ms is very weak. It is not clear why liver homogenates were used and not endo-lysosomal preparations. Does the homogenate contain NAADP-binding protein embedded in the lysosomal membrane? The affinity-labeling data to my mind only show that most labelling was unspecific. Is it possible to co-immunoprecipitate TPC protein with NAADP-labelled protein? If the number of binding sites were not unambigously determined, it is not possible to conclude that the homogenate of wt and DKO liver cells do not differ in their population of NAADP-binding protein. I would recommend to omit the entire para + Figure. It does not help to clarify the controversy.

Concluding Remarks
This ms nicely demonstrates that TPC channels are NAADP-regulated Ca2+-permeable channels in MEFs. Given the importance oft he NAADP-signalling pathway for lysosomal function, the ms would largely benefit from a concise description of the DKO mouse phenotype.

1st Revision - authors' response 12 February 2015

Response to referees' comments

Expert advisor:
'The Ca2+ measurements are solid with one exception. In all traces the NAADP-evoked signal has a sustained elevated response. The question arising is whether this response is mediated by Ca2+ influx. This can easily be tested by measuring the response to NAADP in the absence of external Ca2+. If it is, then the peak and extent of NAADP-evoked Ca2+ release need to be re-evaluated in wild-type, the various knockout and re-expressed conditions by measuring the release in the absence of external Ca2+.

We have now extended the characterization of the NAADP-induced Ca2+ responses in WT MEFs. We confirm that the presence of extracellular Ca2+ is not a confounding factor for our conclusions: although Ca2+ removal unmasked oscillations in some cells, the Maximum Peak amplitude that we routinely measure is unaffected by extracellular Ca2+ removal (new data, Fig 2C, D). This confirms that the early phase is Ca2+ release from intracellular stores only. However, the Mean Ca2+ response is reduced by extracellular Ca2+ removal reflecting recruitment of Ca2+ influx in the later phase.

Since Ca2+ influx plays no role in the early phase of the NAADP response, our existing data (+Ca2+o) with altered TPC expression remain valid and require no repeat in Ca2+-free medium. We present new data that may explain why Ca2+ influx has a role in the *later* phase: Ca2+ release from endo-lysosomal compartments (Fig 2A, B) is amplified via Ca2+ release from the ER (new data, Fig 2E-L). Therefore, recruitment of Ca2+ influx is to be expected as a consequence of (ER) store-operated Ca2+ entry.

I am more concerned about the electrophysiology, in particular the Ca2+ selectivity of the current. It is commendable that the authors measured the native NAADP-activated current and show it to be Ca2+ selective. However, in doing so they use an artificial reconstitution system that may affect channel properties. When measured in lysosomes expressing TPC1 or TPC2 (the native membranes) the current does not show Ca2+ selectivity. I would have liked the authors to have at least expressed TPC1 and TPC2 in their fibroblasts (or COS cells as was done originally) and used their recording system to show that at least one of the channels is indeed Ca2+ selective relative to K+.

We are unsure what the referee means by 'an artificial reconstitution system' because our electrophysiological recordings were not of heterologously expressed proteins, but rather of endogenous currents. We are also confused as to where we show that it is Ca2+ selective, unless the referee means when comparing Ca2+ and K+ ? Results from the electrophysiology experiments we have performed in WT MEFs show that the NAADP-stimulated current displays a rank order of selectivity of Na+ ³ Ca2+ >> K+, therefore, and overall, we do not classify the current as Ca2+ selective.

The advisor suggested that it would be interesting to evaluate the contribution of TPC1 vs TPC2 for the selectivity of Ca2+ over K+ that we observe. To address this question we have analysed NAADP-induced currents in mixed Ca2+/K+ solutions using MEFs from single KO animals (Fig 3A-C), so that only TPC1 or TPC2 is present. Results from TPC1 KO MEFs show no significant differences from WT cells suggesting that TPC2 is Ca2+-selective over K+. In TPC2 KO MEFs currents were too small to be detected; as we highlight in the new Discussion, the lack of TPC1 currents may reflect the fact that our organelle preparation does not contain TPC1-decorated vesicles (small endosomes?). Therefore at this stage is difficult to evaluate TPC1's contribution to the ion selectivity we have observed.

Referee #1:

For the last years there has been a debate as to whether the endosomal/lysosomal TPC channels mediate NAADP-triggered Ca efflux from acidic subcellular compartments. Whilst several groups found strong evidence for this notion by several methods, both the Ca selectivity and the responsiveness to NAADP have been challenged by other labs that used genetically modified cells claimed to be deficient of functional TPC1 and TPC2. Ruas et al now use newly generated knockout mice demonstrably lacking both TPCs. With cells from these as control, the authors reconfirm that TPCs are required for NAADPinduced lysosomal Ca release (live-cell Ca imaging) and for NAADP-induced endosomal/lysosomal Ca currents (planar patch-clamp of vacuolin-enlarged organelles). Importantly, they show that reexpression of TPCs (but not of transport deficient mutants of TPC2) in TPC1/2 double knockout reconstitutes NAADP-induced Ca release - even those truncated versions of TPC1 and 2 that are likely expressed in the mice previously claimed to be knock-out. Finally the authors show by a radioactive binding assay that TPCs themselves are not the NAADP receptors. Overall this is a timely and important study that convincingly reconfirms the proposed function of TPCs as NAADP-triggered Ca release channels and which explains partly some of the reasons for the debate over the last years (previous knockout mice not really being knock-out). The study is clearly described and has been performed with the necessary controls. Several points should be addressed before publication: *Ion selectivity of TPC: The authors tested the Na/Ca permeability ratio only by substituting 'cytosolic' K by Na, but did not test* ***Na on the luminal side****. However, Xu and coworkers postulate that lysosomal exit of Na through TPC2 is important, and luminal Na concentration may be higher than in the cytosol (even though the determination of lysosomal Na by Xu et al. is flawed). It is conceivable that TPCs are more Na- and less Ca-permeable with Na in the inside (or both sides) of the vesicle. This should be checked electrophysiologically.*

© European Molecular Biology Organization

We now provide new data (Fig 4E, F) with Na+ on both sides of the membrane. In mixed (luminal and cytosolic) Ca2+/Na+ solutions the reversal potential is –3.8 ± 2.9 mV, which equates to a permeability ratio 0.86 ± 0.22. This is a very similar value to that obtained using luminal Ca2+ / cytosolic Na+ solutions (Fig 4C, D), reinforcing our conclusion that the permeability of TPCs for these two ions is very similar and that the channel is thus a Ca2+-permeant non-selective ion channel.

Minor points:
- pH measurements were claimed to cover "the entire endo-lysosomal system" (p.7). Most likely the authors measured lysosomal pH, but that is hard to judge because there is no description as to how they did this (loading, chase time, calibration) in the methods. These experimental details should be given.
We apologise for the inadvertent omission. The experimental procedure is now described in detail in the supplementary file.

- Fig.5C: TPC1 only partially co-localizes with LysoTracker, indicating it's early-endosomal. Please comfirm by colocalization with marker, such as EEA1. And please colocalize TPC2 with lamp1, which is more specific and preferable to LysoTracker.
As requested by the reviewer we now include co-localization data obtained from IF images using antibodies against mCherry (mCh) and endogenous organelle marker proteins (new data, Supplementary Fig S6); TPC1.mCh co-localizes mainly with recycling endosomes, as previously observed for mouse TPC1 (Ruas et al 2014), whereas TPC2.mCh localizes not only to lysosomes/late endosomes, but also to recycling endosomes. The observed localization of TPC2.mCh in recycling endosomes (and retention in the ER) is somewhat unexpected, and it could be due to the method of lentiviral delivery of constructs used for expression of cDNAs. Despite the broader pattern of expression of TPC2.mCh in DKO MEFs, the Ca2+ signals evoked by NAADP in these cells retain the expected acidic Ca2+ store pharmacology, i.e. inhibition by bafilomycin A1 and trans-Ned-19 (new data, Fig 5G).

- P4: calling the other groups interrelated seems a bit polemical.
Our intention was to highlight the fact that the same line of *Tpcn1/2* mutant mice was used by both groups. We are sorry if this came across as polemical. As suggested by the reviewer this wording has been modified.

- P4: last sentence: already here mention briefly why it's questionable that the previous mice are indeed knockout.
The whole sentence now reads: However, whether these mice are *bona fide* TPC-null is open to debate as they have the potential to express ≥91% of the full-length TPC sequences.

- P5, first line of results: T159, not T157
This has been corrected.

- P6 and suppl fig S1: do macrophages have only TPC2? Why not even tested for TPC1 in RTPCR?
We have now included RT-qPCR data (new data, Supplementary Fig S2B) showing that macrophages express both *Tpcn1* and *Tpcn2*. Additionally, we show that although *Tpcn1* mRNA is indeed more abundant than *Tpcn2* mRNA, the ratio of *Tpcn1* over *Tpcn2* expression in macrophages (1.7) is not as marked as for other cell types (MEFs: 3.0, new data Fig 1C; liver: 43.9, Fig 8A); whether this is a contributing factor for the strong requirement for *Tpcn2* in NAADP-induced Ca2+ release seen in this cell type is unclear at this stage.

Replace 'knockdown' on page 6 by 'knockout'.
This has been corrected.

- P.17: controversy has (space missing)
This has been corrected.

- Fig.7C: pictures too dark, hard to see anything; in general, immunofluorescence pictures are too small.
In order to highlight the different levels of expression of mCherry-tagged TPCs, we used the same acquisition parameters throughout all panels C in Figs 5–7. This is the reason why the red signal in

© European Molecular Biology Organization

Fig 7C is faint, reflecting the lower level of expression of ΔN-TPCs when compared to the full-length equivalents (Fig 5C); this is in line with results obtained by immunoblotting (Fig 7B). We now include an extra figure (Supplementary Fig S5), showing larger images of panels C with *post hoc* manipulation of the mCherry signal intensity to equalize, as much as possible, the signal in lower expressing cells.

- Legend Fig 1C: better "highlighted with blue bar" (was not easy to see what was meant)
We have changed the figure to make it clearer what the RT-PCR products correspond to. The exons shown correspond to the amplified portions of the cDNAs in the RT-PCR reactions. The legend has been corrected accordingly. Equivalent changes were made in other figures.

- Legend Fig 2A,B: p>0.05 (ns) relative to control: not clear what it relates to and what it means.
We have changed the text to: Control (Ctrl) was pre-incubated with DMSO (vehicle); n = 121–272; $p > 0.05$ (ns) relative to control.

- Possible differences between planar patch clamp used here and by some of the authors before and 'could be conventional' patch-clamp by the "opposing labs" could be discussed.
For our electrophysiological measurements we have used a planar patch-clamp technique where the enlarged endo-lysosomes are pipetted onto a chip containing a micro-aperture for wholeorganelle recording. The Wang *et al* (2012) and Cang *et al* (2013) studies have used a conventional patch clamp technique using a glass micropipette. It remains to be seen if the ability to detect Ca2+ currents is a function of the type of patch-clamp technique used. However, NAADP-regulated currents in enlarged endo-lysosomes preparations have also been observed using the conventional patch-clamp technique (Jha et al 2014). This point is now briefly discussed.

- Please discuss in more detail the recent paper by Jha et al. , in particular whether a difference in Mg concentration can explain the difference between the present work and those studies where no effect of NAADP on TPCs could be found.
Jha et al report that TPC2 is strongly inhibited by Mg2+ ions (Ki 60-130 μM), independently of whether NAADP or PI(3,5)P2 was the stimulus. It remains to be seen whether Mg2+ is a universal TPC inhibitor, (sea urchin egg homogenate responds robustly to NAADP with a free Mg2+ concentration > 1 mM). Nonetheless, it is interesting to note that the vast majority of studies reporting successful activation by NAADP, do so in the absence of Mg2+, while Wang *et al* 2012 and Cang *et al* 2013 include inhibitory (mM) concentrations of Mg2+ in their experimental conditions. However, Mg2+ cannot conveniently explain their lack of an NAADP response: millimolar Mg2+ should also inhibit PI(3,5)P2-stimulated currents (according to Jha 2014) and yet Wang et al still observe a robust stimulation.
We hope that the referee agrees with us that an extended discussion of these points is too speculative (because there are too many unknowns) and would only serve to dilute the salient discussion. We would therefore prefer to keep the point as brief as possible.

- Could the authors perform Western blots to additionally confirm the present KO mouse lines?
The mouse lines we have used in the crosses to produce the *Tpcn1/2*—/— line have been characterized previously. In Ruas et al 2014 describing the *Tpcn1*—/— line we show lack of TPC1 protein in a variety of tissues, in addition to lack of *Tpcn1* cDNA. We have also shown lack of *Tpcn2* cDNA in liver from *Tpcn2*—/— mice (Calcraft et al 2009) and in a variety of other tissues (unpublished; see below).

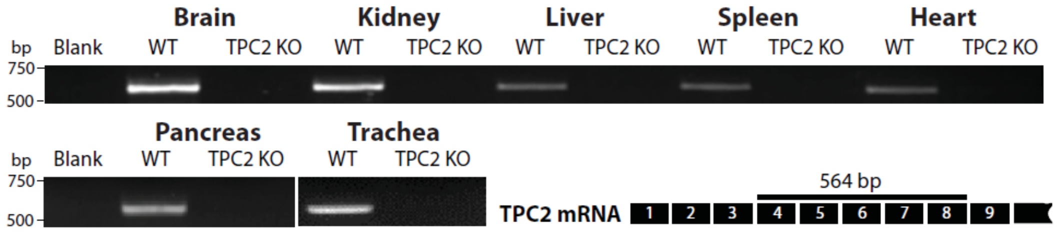

Unfortunately, in spite of our best efforts, we have not been able to obtain convincing immunoblots using several of the anti-TPC2 antibodies we tested, possibly due to the poor ability of the antibodies to detect low endogenous levels of TPC2 protein.

We hope however, that the reviewer will agree with the fact that if no *Tpcn1* or *Tpcn2* mRNAs are detected in samples from *Tpcn1/2*—/—, no equivalent proteins are expected to be present.

- Western in Fig. 7B: I am puzzled by the large apparent size difference between TPC1 FL and TPC1 deltaN69. Further, please include a non- or mock-transfected control and run the samples on the same gel (the present one is cut and put together).
We have noticed that the lanes from the original figure had been mislabelled, for which we apologize and are grateful that the discrepancy was noted. We would prefer to keep the corrected panel in the main figure, as the samples were derived from the same batch of cells used in the experiments shown in the other panels. The immunoblot corresponds to a single gel, although the order of the lanes has been rearranged; unfortunately, on that gel we did not include a mock-transfected control.

We now show a new set of immunoblots (new data, Fig S6) where we include samples from mocktransfected cells.

TPCs are glycosylated proteins and in agreement with our recent publication (Ruas et al 2014) we have noticed that ΔN-TPCs show a reduced level (sometimes undetectable levels) of heavily glycosylated forms compared to the core-glycosylated form. This can exacerbate the difference in the electrophoretic mobility between FL- and ΔN-TPCs. To better compare the sizes of FL- and ΔNTPCs we have performed immunoblots using PNGase F-treated samples in order to de-glycosylate the proteins and reveal the core peptide mobility (new data, Fig S6).

- Ca-signal in Fig. 7E: the increase of calcium concentration with the TPC1 deltaN69 is much delayed. Is this typical? Do you think it results from Ca-induced Ca-release from ER stores?
We have measured the lag time (time from addition of NAADP/AM to initiation of Ca2+ rise; excluding non-responders) and the results show no significant differences between the two sets of cells: TPC1- FL = 49 s ± 3 (n=174), TPC1-ΔN69 = 46 s ± 3 (n=118). Therefore, we have selected a different trace for TPC1 ΔN69 in Fig 7E, so as not to mislead readers.
We also include new data in Fig 2 showing that the NAADP-induced Ca2+ signals in MEFs are amplified via CICR.

Referee #2:

NAADP induced Ca2+ release from lysosomal stores represents an important lysosomal signaling pathway. Two-pore channel proteins TPC1 and TPC2 localized at the intracellular endosomes and lysosomes (‚endo-lysosomes') were proposed to be important players in NAADP-triggered endolysosomal Ca2+ release. However, conflicting findings were reported concerning NAADP-sensitivity (and ion selectivity) of TPC channels. Importantly, a recent publication by Wang et al. in Cell reported that TPC channels are insensitive to NAADP and that TPC-mediated currents are selective for Na+, challenging the role of TPC channels as NAADP-regulated Ca2+-permeable ion channels. Here, Ruas et al., taking advantage of a TPC1/2 double knock-out (DKO) mouse line, have characterized TPC1/2 channel properties in endo-lysosomes, and come to very different conclusions. Based on their results Ruas et al. propose that TPC channels are Ca2+-permeable ion channels indispensible for NAADP signalling. Ruas et al. tested the ability of DKO mouse embryonic fibroblasts (MEFs) to respond to NAADP. The data demonstrates that DKO MEF showed no Ca2+ response to NAADP. Measurements of TPC channel activity are technically very challenging and mostly rely on isolated vacuolin-1 swollen lysosomes, a procedure with many pitfalls and experimental artefacts. Importantly, Ruas et al. could rescue NAADP-induced Ca2+ release in DKO MEFs by lentivirus-based reexpression of mCherry tagged TPC1 and TPC2, whereas expression of TPC pore mutants failed to rescue NAADP-induced Ca2+ release. Also, the authors carried out planar-patch clamp experiments on swollen endolysosomes to investigate NAADP-sensitivity and ion selectivity. The data indicates that NAADPstimulated currents displayed a rank order of selectivity of Na+ {greater than or equal to} Ca2+ >> K+. These results demonstrate that in the absence of Na+, Ca2+ is the major cation permeating TPC channels. In a final chapter Ruas et al. address the possibility that NAADP does not directly bind to TPC channels, but binds to an accessory protein. Based on NAADP binding and affinity labeling experiments it was concluded that NAADP does not bind directly to TPC channels and that an auxiliary NAADP-binding protein confers NAADP regulation.

Specific comments:
In Discussion Ruas et al. are mainly concerned with studies of competitors who challenged the view that TPC channels are NAADP-regulated Ca2+ premeable channels. The Discussion section is as along as the results section and should significantly be shortened. It reiterates many points that were already made in Results and need not be repeated. It suffices to state the differences, e.g. the previous mice were not null for TPC, whereas the present mice were definitely null. Furthermore, it should be emphasized that besides differing experimental conditions (overexpression in tissue culture cells) different preparations were used. Assuming that the NAADP binding protein is an accessory protein, it may not be expressed in all cells alike, e.g. HEK293, COS-1, macrophages, and MEFs. This point may need more emphasis than others, which should be discussed in a much more concise manner. In this context the authors should also comment on the fact that they measured currents in the range of 10 - 20 pA, whereas the competitors reported currents in the 1000 - 2000 pA range. This difference may have quite an impact on the interpretation of some of the electrophysiological data.
We have simplified the discussion as suggested and emphasized certain areas that benefited from a more in-depth discussion. However, it is unlikely that the lack of NAADP-induced responses in Wang *et al* (2012) and Cang *et al* (2013) can be explained by the use of different cell types/lines, as suggested by the reviewer: HEK293 and COS cells have been used in several studies reporting NAADP-induced responses (for example, Calcraft *et al* 2009, Ruas *et al* 2010, Jha *et al* 2014) and in this manuscript we show that macrophages are also able to promote NAADP-induced Ca2+ signals contrary to previous reports.

We also now briefly discuss the amplitude ranges, in line with referee's good suggestion.

Fig. 4: It would be nice to show one experiment in the absence of Ca2+ in a Na+ only configuration similar to the conditions of Wang et al. (2012). Also, it would be helpful to include a current trace, where PI(3,5)P2 and NAADP were added together.
Our results using bi-ionic conditions with luminal Ca2+ / cytosolic Na+ show that the NAADPstimulated current shows a PCa/PNa permeability ratio of 0.57 ± 0.19. Unfortunately, we are not technically able to calculate permeability ratios with the ionic gradient reversed (luminal Na+, cytosolic Ca2+) because the planar patch-clamp technique has an absolute requirement for luminal Ca2+ for seal formation. As an alternative, we have recorded currents with both Na+ and Ca2+ competing in the lumen; the PCa/PNa permeability ratio was not affected under these conditions (new data, Fig 4E, F). We hope this is acceptable to the referee.
The question of modulation of NAADP responses by PI(3,5)P2, first demonstrated in Jha *et al* (2014), is certainly a very interesting one. We plan to study this modulation in detail and the results obtained will be reserved for such a study. In our opinion results from this experiment would not greatly strengthen the take-home message of this manuscript.

Fig. 8: NAADP-binding studies.This part of the ms is very weak. It is not clear why liver homogenates were used and not endo-lysosomal preparations. Does the homogenate contain NAADP-binding protein embedded in the lysosomal membrane? The affinity-labeling data to my mind only show that most labelling was unspecific. Is it possible to co-immunoprecipitate TPC protein with NAADP-labelled protein? If the number of binding sites were not unambigously determined, it is not possible to conclude that the homogenate of wt and DKO liver cells do not differ in their population of NAADP-binding protein.
I would recommend to omit the entire para + Figure. It does not help to clarify the controversy.
The identification of the NAADP-binding protein is at present a matter of intense research and we are engaged in identifying such a protein responsible for NAADP-regulation of Ca2+ release. We have previously shown that immunoprecipitates from endogenous sea urchin TPCs contain NAADPbinding proteins (Ruas *et al* 2010, Walseth *et al* 2012a). We have employed a similar strategy for mouse samples, but unfortunately we have not been able to see co-immunoprecipitation of NAADPbinding proteins; this could either be due to inability of the anti-TPC antibodies to coimmunoprecipitate interacting proteins, or/and nature of interaction between TPC and associated proteins.
Following from our previous studies, a question that is important to answer is: are TPCs themselves the direct binding target for NAADP? In our opinion the critical experiment related to this question is the study of NAADP-binding properties in a TPC-null system, such as the one here described and therefore we argue that data related to this question should be included in the manuscript; this has

© European Molecular Biology Organization

also been recommended by the editor evaluating the manuscript submission. However, we would argue that the description of the nature of the NAADP-binding protein and further characterization is more appropriate for a further manuscript.

Concluding Remarks: This ms nicely demonstrates that TPC channels are NAADP-regulated Ca2+-permeable channels in MEFs. Given the importance of the NAADP-signalling pathway for lysosomal function, the ms would largely benefit from a concise description of the DKO mouse phenotype.

Since the development of our *Tpcn1/2–/–* mouse line we have been involved in several collaborative studies dealing with physiological pathways where TPC function might be important. The description of phenotypes observed in *Tpcn1/2–/–* mice are being published as part of those studies, such as the one recently published (Lear *et al*. Absence of Intracellular Ion Channels TPC1 and 2 Leads to Mature-Onset Obesity in Male Mice, Due to Impaired Lipid Availability for Thermogenesis in Brown Adipose Tissue. Endocrinology. 2014 Dec 29:en20141766. [Epub ahead of print]).

2nd Editorial Decision | 03 March 2015

Thank you for submitting a revised version of your manuscript to The EMBO Journal, it has now been by the two original referees whose comments are shown below. As you will see they both find that the original concerns have been sufficiently addressed and recommend publication in The EMBO Journal.

Thank you again for submitting your work for The EMBO Journal, I look forward to receiving the final revision.

REFEREE REPORTS

Referee #1:

The authors did a good job in revising their manuscript, and almost all my points have been addressed in a satisfactory manner. It is a bit unfortunate that they cannot prove that they have a full KO of both TPCs also by Western blot analysis because the lack good antibodies. Nonetheless, this important work seems to be ready for publication.

A very minor point is that it remains unclear what the p>0.05 (ns) refers to in the legend to figure 2 A,B, as no ns is indicated in the figure.

Referee #2:

Ruas and collaborators have added essential experiments and additional information to their revised manuscript version. The authors have adequately dealt with most queries raised by the reviewers. Though a convincing experiment about the nature of the NAADP binding protein is still at large, I recommend to publish the revised ms of Ruas et al in EMBO Journal.
